# NK-92MI Cells Engineered with Anti-claudin-6 Chimeric Antigen Receptors in Immunotherapy for Ovarian Cancer

**DOI:** 10.7150/ijbs.88539

**Published:** 2024-02-11

**Authors:** Junping Li, Hong Hu, Hui Lian, Shuo Yang, Manting Liu, Jinping He, Bihui Cao, Dongni Chen, Yuling Hu, Chen Zhi, Yan Shen, Xiaodie Ye, Bingjia He, Ming Zhao, Weijun Fan, Linfeng Xu, Rom Leidner, Qingde Wu, Lili Yang, Zhenfeng Zhang

**Affiliations:** 1 Department of Radiology; Translational Medicine Center; Guangzhou Key Laboratory for Research and Development of Nano-Biomedical Technology for Diagnosis and Therapy & Guangdong Provincial Education Department Key Laboratory of Nano-Immunoregulation Tumour Microenvironment; Central Laboratory, the Second Affiliated Hospital of Guangzhou Medical University, Guangzhou 510260, China.; 2 Department of Radiology, Xiangyang No.1 People's Hospital, Hubei University of Medicine, Xiangyang 441000, China.; 3 Department of Nutrition; Guangdong Provincial Key Laboratory of Food, School of Public Health, Sun Yat-sen University, Guangzhou 510080, China.; 4 Department of Radiology, Shunde Chinese Medicine Hospital, the Affiliated Hospital of Traditional Chinese Medicine University of Guangzhou, Foshan 528000, China.; 5 Guangzhou Municipal and Guangdong Provincial Key Laboratory of Molecular Target & Clinical Pharmacology, The NMPA and State Key Laboratory of Respiratory Disease, School of Pharmaceutical Sciences and The Fifth Affiliated Hospital, Guangzhou Medical University, Guangzhou, 510260, China.; 6 Department of Pathology, the Second Affiliated Hospital of Guangzhou Medical University, Guangzhou 510260, China.; 7 Minimally Invasive Interventional Division; State Key Laboratory of Oncology in South China; Collaborative Innovation Center for Cancer Medicine, Sun Yat-sen University Cancer Center, Guangzhou 510060, China.; 8 Department of Interventional Therapy, Sun Yat-Sen Memorial Hospital, Sun Yat-Sen University, Guangzhou 510260, China.; 9 Earle A. Chiles Research Institute, Providence Cancer Institute, 4805 NE Glisan St., Suite 2N35, Portland, OR 97213, USA.

**Keywords:** ovarian cancer, chimeric antigen receptor, NK cells, Claudin-6, PD-L1, PD-1

## Abstract

**Background**: The application of chimeric antigen receptor (CAR) NK cells in solid tumors is hindered by lack of tumor-specific targets and inefficient CAR-NK cell efficacy. Claudin-6 (CLDN6) has been reported to be overexpressed in ovarian cancer and may be an attractive target for CAR-NK cells immunotherapy. However, the feasibility of using anti-CLDN6 CAR-NK cells to treat ovarian cancer remains to be explored.

**Methods**: CLDN6 expression in primary human ovarian cancer, normal tissues and cell lines were detected by immunohistochemistry and western blot. Two types of third-generation CAR NK-92MI cells targeting CLDN6, CLDN6-CAR1 NK-92MI cells with domains containing self-activated elements (NKG2D, 2B4) and CLDN6-CAR2 NK-92MI cells with classical domains (CD28, 4-1BB) were constructed by lentivirus transfection, sorted by flow cytometry and verified by western blot and qPCR. OVCAR-3, SK-OV-3, A2780, Hey and PC-3 cells expressing the GFP and luciferase genes were transduced. Subcutaneous and intraperitoneal tumor models were established via NSG mice. The ability of CLDN6-CAR NK cells to kill CLDN6-positive ovarian cancer cells were evaluated in vitro and in vivo by live cell imaging and bioluminescence imaging.

**Results**: Both CLDN6-CAR1 and CLDN6-CAR2 NK-92MI cells could specifically killed CLDN6-positive ovarian cancer cells (OVCAR-3, SK-OV-3, A2780 and Hey), rather than CLDN6 negative cell (PC-3), in vitro. CLDN6-CAR1 NK-92MI cells with domains containing self-activated elements (NKG2D, 2B4) exhibited stronger cytotoxicity than CLDN6-CAR2 NK-92MI cells with classical domains (CD28, 4-1BB). Furthermore, CLDN6-CAR1 NK cells could effectively eliminate ovarian cancer cells in subcutaneous and intraperitoneal tumor models. More importantly, CAR-NK cells combined with immune checkpoint inhibitors, anti-PD-L1, could synergistically enhance the antitumor efficacy of CLDN6-targeted CAR-NK cells.

**Conclusions**: These results indicate that CLDN6-CAR NK cells possess strong antitumor activity and represent a promising immunotherapeutic modality for ovarian cancer.

## Background

Ovarian cancer (OC) is a common gynecological malignant tumor and one of the main causes of cancer-related death in women^1^. It is estimated that by 2035, the incidence and mortality of OC will increase globally^2^. Currently, as many as 80% of patients relapse within two years after surgery and traditional chemotherapy and eventually develop chemo-resistance, with a 5-year survival rate of only 40-45%^3^, ^4^. The recently developed molecular targeted drugs and immune checkpoint inhibitors do not show obvious advantages over traditional chemotherapy^5^-^7^. Therefore, there is an urgent need for new effective treatment strategies for OC.

At present, chimeric antigen receptor (CAR)-modified T or NK cell (CAR-T/NK) therapy has been proven to be an effective tumor therapy, especially the successful application of CD19-targeted CAR-T/NK cell therapy in certain leukemia and lymphoma^8^, ^9^. CAR-NK cells are relatively safer in treatment without cytokine release syndrome (CRS) or graft-versus-host disease (GVHD), which is becoming a new research hotspot in the field of tumor immunotherapy^9^. CAR-NKs not only inherit the idea of classical CAR-T cells (T cell activation receptor CD28, 4-1BB as domain), but many innovations and developments based on the biological characteristics of NK cells (NK cell activation receptor NKG2D, 2B4 as domain) have also been realized^10^-^12^. CAR-NK cells also express a variety of activated receptors, which can specifically recognize the ligands expressed on tumor cells and improve the effect of immunotherapy^13^. To improve the therapeutic effect of CAR-T/NK cells, it is obvious that the structural design of CAR molecules also needs continuous innovation^14^.

CAR-NK transmembrane structures are most commonly derived from CD3ζ, CD8, or CD28^15^. Some studies have also explored CD16, NKp44, NKp46, or NKG2D as transmembrane domains for the CAR-NK^14^. Interestingly, NK cell-activated receptors as transmembrane structures can lead to more CD107a degranulation and stronger cytotoxicity^16^. The origin of the transmembrane structure plays a decisive role in the activity of CAR-NK cells. NKG2D is an important activated receptor of NK cells that can bind to the transporters DAP10 or DAP12 to activate the cytotoxicity of NK cells^12^. Therefore, as a transmembrane structure of NK cells, NKG2D has innate advantages. CD28, 4-1BB, OX40 and 2B4 are common intracellular costimulatory domain structures. At present, most of the CAR costimulatory domains are based on CD28 or/and 4-1BB of T cells and many related clinical investigations have been achieved, which belong to the classical T cell CAR molecular domain^9^, ^14^, ^17^. 2B4 is an important activation signal regulator of NK cells that contains a cytoplasmic domain and an immune receptor tyrosine-based switching motif (commonly known as ITSM), which is responsible for interacting with multiple signal adapters and transmitting activation signals^11^. In addition, the intracellular signal domains of DAP10 and DAP12 based on the characteristics of the intracellular activation pathway of NK cells showed the role of inducing the activation of NK cells^18^. Recent studies on CAR-NK cells have also confirmed the key role of DAP10 or DAP12 in intracellular signal transduction^14^. Therefore, the selection of intracellular costimulatory activation signals will also have a significant impact on the function and persistence of CAR-NK cells. NKG2D, 2B4 and DAP10 have roles in signal transduction and the function of inducing NK cell activation^14^, ^18^. Therefore, in this study, we constructed a novel third-generation CAR-NK containing the NKG2D transmembrane domain, the 2B4 and DAP10 intracellular domains (CAR_1_) and a traditional third-generation CAR-NK containing the CD28 transmembrane domain, the CD28 and 4-1BB intracellular domain (CAR_2_) and conducted a comparative study on the biological functions of the two. Although CAR-T/NK cells show good clinical effects in patients with malignant hematological diseases, there are still many challenges in the treatment of solid tumors, such as finding safe and effective solid tumor antigens and maintaining the persistence of CAR cells in the tumor microenvironment^19^.

The main challenge of CAR immunotherapy for ovarian cancer is the selection of a specific antigen to distinguish tumors from normal tissue^20^. Ideally, CAR-targeting molecules should be highly expressed in tumor tissue, with no or very low expression in normal cells^21^. In recent years, Claudin-6 (CLDN6), one of the 27 members of the Claudin family, has been widely studied by researchers^22^. Claudin-6 is a cell surface membrane protein that is highly expressed in various solid tumors, such as ovarian cancer, testicular cancer and endometrial cancer^23^. CLDN6 is involved in regulating the proliferation and apoptosis of cancer cells through different pathways, leading to the procancer or anticancer effects of CLDN6 in different cancers^24^. Clinical trials of IMAB027 (NCT02054351) and ASP1650 (NCT03760081) (http://clinicaltrials.gov) developed with Claudin-6 as the target have been carried out in ovarian cancer, germinoma and other malignant tumors^25^. Antibodies against CLDN6 have also been designed as bispecific antibodies and cytotoxic drug conjugates, and all of them may have good antitumor effects^23^, ^26^, ^27^. A large number of studies have shown that CLDN6 is not expressed in normal adult tissues but is expressed on the surface of a variety of cancer cells, which is an ideal target for tumor therapy^22^, ^28^. In 2020, second-generation CLDN6 CAR T cell expansion driven by an RNA vaccine was reported in Science and achieved good preclinical results^28^. However, the feasibility and efficacy of third-generation CAR-NK cells targeting CLDN6 in the treatment of ovarian cancer has not been evaluated.

In addition, ovarian cancer is an immunosuppressive tumor, and the effect of immune checkpoint inhibitors in ovarian cancer is limited, while the tumor microenvironment inhibits the function of immune cells^29^, ^30^, so innovative immunotherapy is urgently needed. A large number of studies have shown that PD-1 can also be expressed by NK cells in cancer patients, including ovarian cancer patients^31^-^33^. Previously, antibodies against PD-1 and PD-L1 were thought to benefit only T-cell-driven responses; however, blocking the PD-1/PD-L1 axis may also improve NK cell therapy through an indirect and important mechanism^34^.

In this study, we first determined that CLDN6 is an effective target for ovarian cancer and then constructed two types of third-generation CAR-NK cells with different domains targeting CLDN6 to explore their antitumor activity in vivo and in vitro and the effect of regional cell therapy in vivo and to observe the biological characteristics of CAR-NK cells by real-time dynamic imaging of living cells. We also explored a new combination of immunotherapy, CAR-NK cells combined with immune checkpoint inhibitors, to find a potential combination mechanism to improve the function of CAR-NK cells, hoping to provide a new and effective treatment strategy for clinical treatment in the future.

## Materials and methods

**Cell lines and cell culture**—Human NK-92MI cells were cultured in alpha minimum essential medium (Gibco) with 0.2 mM inositol (Solarbio), 0.1 mM β-mercaptoethanol (Sigma-Aldrich), 0.02 mM folic acid (Sigma-Aldrich), 100 IU/mL penicillin and 100 IU/mL streptomycin (Gibco) and then adjusted to a final concentration of 12.5% horse serum (Gibco) and 12.5% FBS (Gibco). Human NK-92 cells were irradiated with 10 Gy (Biobeam 2000 device, Germany) on the basis of a previous description^35^. SK-OV-3, OVCAR-3, A2780, Hey, PEO4, HO-8910, OV-1063, and PC-3 cell lines were cultured in RPMI-1640 (Gibco) supplemented with 10% FBS (Gibco), 2 mM GlutaMax, 100 IU/mL penicillin and 100 IU/mL streptomycin (Gibco). SK-OV-3, OVCAR-3, A2780, Hey, and PC-3 cells were lentivirally transduced with LV5-LUC-GFP-Puro virus (Genepharma, Shanghai, China) expressing GFP-luciferase (GL) genes. All cell lines were routinely tested for mycoplasma.

**Human samples**—All human sample collection was approved by the Institutional Review Boards of the Second Affiliated Hospital of Guangzhou Medical University, and informed consent was obtained from patients.

**Mouse studies**—Four- to five-week-old female NSG (NOD-Prkdc^scid^ IL2rg^null^) mice were purchased from Biocytogen, Beijing, China. All mice were housed under specific pathogen-free conditions and were provided autoclaved food and water in the Animal Core Facility of Guangzhou Medical University (Guangzhou, China). All mouse experiments were performed in accordance with Guangzhou Medical University Experimental Animal Center and Institutional Animal Care and Use Committee (IACUC) guidelines and were approved by Guangzhou Medical University IACUC.

**Construction of chimeric antigen receptors (CAR)**—The human CLDN6-specific single chain variable fragment (scFv) was derived from the IMAB206-SUBS antibody (WO2012156018A1)^28^. CLDN6-CAR_1_ possessed a human CD8α signal peptide (UniProt: P01732, amino acids [aa] 1-21), human CLDN6 scFv, humanCD8α hinge (UniProt: P01732, aa138-184), human NKG2D (UniProt: P26718, aa52-72), human 2B4 (UniProt: Q9BZW8, aa251-370), human DAP10 (UniProt: Q9UBK5, aa70-93), and CD3ζ (UniProt: P20963, aa52-164) and was linked by a P2A ribosomal skip element to eGFP. The CD19-CAR was based on the same third-generation CAR scaffold with substituted scFv derived from the FMC63 monoclonal antibodies (GenBank: ADM64594.1)^36^. CLDN6-CAR_2_ possessed a human CD8α signal peptide (UniProt: P01732, aa1-21), human CLDN6 scFv, humanCD8α hinge (UniProt: P01732, aa138-184), human CD28 transmembrane (UniProt: P10747, aa114-179), human CD28 (UniProt: P10747, aa180-220), human 4-1BB (UniProt: Q07011, aa214-255), and CD3ζ (UniProt: P20963, aa52-164) was linked by a P2A ribosomal skip element to eGFP. The CD19-CAR_2_ containing the same third-generation CAR_2_ scaffold has been verified in previous experiments^37^, so it will not be repeated in subsequent experiments. The optimized and synthesized codon sequences (GenScript) were cloned into pUC57. The CLDN6-CAR_1_, CLDN6-CAR_2_, and CD19-CAR cassettes were generated by gene synthesis and cloned into the lentiviral vector pWPXLd-2A-eGFP by Genscript Co., Ltd. (Nanjing, China). Gene expression in NK-92MI cells was verified by qPCR and western blotting.

**Lentivirus production and generation of CLDN6-CAR NK92MI cells**—293T cells were transfected with three different plasmids, the pWPXLd-based lentiviral plasmid, together with two auxiliary packaging plasmids, psPAX2 and pMD.2G, using polyethyleneimine transfection (Sigma-Aldrich). Lentiviral supernatants were produced in 293T cells. Supernatants were harvested at 48 and 72 hr post transfection. NK-92MI cells were transduced by filtering lentiviral supernatants with polybrene (8 µg/mL; Sigma-Aldrich) and expanded in NK-92MI special culture medium (Procell). Eight to 12 hours later, NK-92MI cells were examined for growth status, viral supernatant was replaced with fresh medium, and NK-92MI cells were further expanded with half media changes every 48 hours. Seventy-two hours after transduction, NK-92MI transduction was measured by flow cytometry with the marker eGFP. On days 9-10, NK-92MI cells were sorted by fluorescence-activating cell sorting (FACS) for enrichment with the eGFP marker. The sorted NK-92MI cells were subsequently purified 2-3 times by FACS sorting.

**Flow cytometry**—To evaluate CAR or GL expression in transduced cells, flow cytometry detections for CD19- and CLDN6-CAR NK-92MI cells, along with SK-OV-3 GL, OVCAR-3 GL, A2780 GL, PC-3 GL and Hey GL cells, were performed on a Beckman flow cytometer with eGFP as the marker. Transduced CAR-NK-92MI or GL cells were sorted by a FACS sorter (Beckman Coulter MoFlo XDP, USA). We performed flow cytometry using Abs specific to human PD-1, PD-L1 and CD107a (from Biolegend) conjugated with APC fluorochromes. For surface staining, cells were incubated at 4°C for 30 min in staining buffer (PBS, 2% FBS). PB, spleen (SP), and tumor samples from mouse xenografts were treated with red blood cell lysis buffer (Biolegend) and accutase (Gibco), and the single-cell suspension was filtered through a 40 µm filter and prepared for downstream analysis. Subsequently, eGFP was used as a marker and flow cytometry was used to detect the number of infiltrated CAR-NK cells. Samples were acquired with Beckman FACS using CytExpert software. For each sample, a minimum of 10,000 events were acquired, and all data were analyzed by FlowJo 10 software. For all experiments, CAR-NK cells were FACS sorted to >99% purity.

**Western blot**—For Western blot (WB) analysis, cells were washed with PBS and lysed with Laemmli buffer (Bio-Rad). Protein lysates were normalized according to the amount of protein molecules and resolved on 8% - 12% SDS polyacrylamide gel electrophoresis gels (SDS-PAGE, Bio-Rad). After protein transfer to polyvinylidene fluoride (PVDF) membranes (Merck Millipore), the membranes were blocked in 5% nonfat milk in Tris-buffered saline Tween (TBST) and incubated with primary and secondary antibodies (Abs) in TBST with 1% nonfat milk. Subsequent procedures were modified from the standard protocol. Membranes were probed with rabbit anti-human CLDN6 primary antibody (Abcam), anti-human PD-L1 primary antibody (CST), or anti-human CD3 (Abcam) primary antibody. Membranes were developed with SuperSignal West Pico PLUS Chemiluminescent Substrate (ThermoFisher Scientific) by a gel imaging analysis system (Bio-Rad).

**Immunohistochemistry**—To examine CLDN6 protein expression in human ovarian cancer and normal tissues, an immunohistochemistry (IHC) staining assay was performed. All human sample collection was approved by the Institutional Review Boards of the Second Affiliated Hospital of Guangzhou Medical University. Formalin-fixed paraffin-embedded tissues were sectioned at a thickness of 4 μm, baked for 1 hr at 60°C, dewaxed, hydrated, antigen repaired, blocked for endogenous peroxidase activity, blocked, incubated with primary and secondary antibodies, and stained using a standard hematoxylin and eosintechnique. Paraffin sections were also immunostained with a primary antibody specific for CLDN6 (Abcam) at 1:100 overnight at 4 °C, followed by secondary antibody staining with goat anti-rabbit IgG HRP (MXBbiotechnologies, China). Staining was visualized using peroxidase streptavidin (MXB biotechnologies, China) for 10 min, followed by 3,3'diaminobenzidine (DAB, MXB). Images of all slides were obtained with an Olympus TH4-200 microscope or 3DHISTECH's slide converter. Different expression levels of CLDN6 in 62 ovarian cancer samples and 11 important organs of the human body were evaluated by two experienced pathologists using a 4-point scale in a blinded fashion. A score of 0 indicates no CLDN6 expression; scores of 1+, 2++, and 3+++ indicate weak to strong expression of CLDN6. For the tumor samples from mouse xenografts, paraffin sections were incubated with antibodies specific for CD3 (Abcam) overnight at 4 °C, followed by secondary antibody with goat anti-rabbit IgG (H + L) (Beyotime). Images of sections were obtained with a microscope (Leica DFC7000 T). The percentages of CLDN6- and CD3-positive staining with different scores were recorded and analyzed by ImageJ software.

**Quantitative real-time polymerase chain reaction (qPCR) and agarose gel electrophoresis (AGE)**—To examine the expression of CLDN6 in ovarian cancer cells and different CAR molecules in CAR-NK cells, total RNA was extracted by TRIzol reagent (Sigma-Aldrich). cDNA was generated using the Fast All-in-one RT kit (with gDNA Remover) (ESscience biltech) or HiScript III RT SuperMix for qPCR (+gDNA wiper) (Vazyme) according to the manufacturer's instructions. mRNA expression was detected via qPCR with ChamQ Universal SYBR qPCR Master Mix (Vazyme) according to the manufacturer's instructions. The glyceraldehyde-3-phosphate dehydrogenase (GAPDH) gene was used as an endogenous control. The expression of CLDN6 and CARs was analyzed in triplicate and normalized to GAPDH. All data were analyzed by QuantStudio™ Real-Time PCR Software. To identify CAR molecular expression, AGE was used to detect CAR-NK RT-PCR products with water as a negative control and vector as a positive control. After digestion of the CAR lentiviral vector plasmid by the restriction endonucleases PmeI and SpeI, the CAR genes were detected by AGE. These primers are detailed below:

GAPDH-Forward Primer: 5'-AGAAGGCTGGGGCTCATTTG-3'; GAPDH-Reverse Primer: 5'-AGGGGCCATCCACAGTCTTC-3'; scFv of CLDN6-CAR1 NK-Forward Primer: 5'-CTACAACGGCGGCACCATCTAC-3'; scFv of CLDN6-CAR1 NK-Reverse Primer: 5'-GGGCACAGTAGTACACAGCAGAATC-3'; scFv of CLDN6-CAR2 NK-Forward Primer: 5'-TACAACGGCGGCACAATCTATAACC-3'; scFv of CLDN6-CAR2 NK-Reverse Primer: 5'-CATGTAGGCTGTACTGCTGCTCTTG-3'; scFv of CD19-CAR NK-Forward Primer: 5'-CTGGAATGGCTGGGCGTTATCTG-3'; scFv of CD19-CAR NK-Reverse Primer: 5'-CCTTGATGATGGTCAGTCTGCTCTTC-3'; CLDN6-Forward Primer: 5'-CCATCATCCGGGACTTCTATAA-3'; CLDN6-Reverse Primer: 5'-CAGACGTAATTCTTGGTAGGGT-3'. The cycle threshold (Ct) was determined using QuantStudio™ Real-Time PCR Software, and the level of gene expression was calculated using the comparative Ct method (2(^Ct)).

**Cytokine release assays**—Enzyme-linked immunosorbent assay (ELISA) kits for Perforin, Granzyme B, Interferon-γ (IFN-γ), Tumor necrosis factor-α (TNF-α) and Granulocyte-macrophage colony stimulating factor (GM-CSF) were purchased from R&D Systems, and all ELISA kits were used according to the manufacturer's protocols. Target cells (1×10^4^) were incubated with effector cells (1×10^4^) in 96-well plates for 24 hours. The culture supernatants were then collected and analyzed with a multifunctional enzyme marking instrument (BioTek, USA).

**Cytotoxicity assays in vitro**—SK-OV-3 GL, OVCAR-3 GL, A2780 GL, Hey GL and PC-3 GL target cells were incubated with parental NK-92MI, CD19-CAR NK, CLDN6-CAR_1_ NK and CLDN6-CAR_2_ NK effector cells at the indicated effector to target (E:T) ratios of 1:2, 1:1, 2:1, and 4:1 in triplicate wells of white 96-well plates. Target cell viability was monitored 8 hours later by adding 100 μl/well D-Luciferin (sodium salt; Yeasen BioTechnology) at 150 μg/mL. Then, the percent viability (%) was computed as experimental signal/maximal signal × 100, and the cell lysis percentage was calculated using 100% - percent viability (%).

SK-OV-3 GL, SK-OV-3 GL with IFN-γ pretreated (IFN-γ-SK-OV-3 GL), OVCAR-3 GL, OVCAR-3 GL with IFN-γ pretreated (IFN-γ-OVCAR-3 GL) target cells were incubated with CLDN6-CAR_1_ NK effector cells at the indicated effector to target (E:T) ratios of 1:2, 1:1, and 1:2 in triplicate wells of white 96-well plates. Target cell viability was monitored 8 hours later and computed following the previously described studies. SK-OV-3 GL, IFN-γ-SK-OV-3 GL, OVCAR-3 GL, and IFN-γ-OVCAR-3 GL target cells were incubated with CLDN6-CAR_1_ NK effector cells at the indicated effector to target (E:T) ratios of 1:1 with or without 10 μg/mL anti-PD-1/PD-L1 in triplicate wells of white 96-well plates. Target cell viability was monitored 8 hours later and computed following the previously described studies. Hey GL target cells were incubated with CLDN6-CAR_1_ NK effector cells at the indicated effector to target (E:T) ratios of 1:1 with or without 10 μg/mL anti-PD-L1 in triplicate wells of white 96-well plates. Target cell viability was monitored (every 2 hours, 6 times in total) and computed following the previously described studies.

**Live cell imaging for the cytotoxicity assay**—SK-OV-3 or PC-3 cells were seeded on gelatin-coated coverslips and placed in the cell culture incubator for 12 hrs to allow cells to adhere and spread on the surfaces. Then, the SK-OV-3 or PC-3 cells on coverslips were cocultured with NK or various CAR-NK cells in the cell culture incubator and loaded in a magnetic chamber (Invitrogen, Thermo Fisher Scientific) for live cell imaging. The magnetic chamber was installed on a microscope stage equipped with an incubator system (EVOS M7000), which could maintain the cell culture conditions (37°C, 5% CO2). After the addition of CAR-NK cells, time-lapse imaging was initiated. Differential interference contrast (DIC) images were acquired every 1 min for 4 h. An Invitrogen EVOS M7000 3D Digital confocal Living Cell Imaging analysis system (Invitrogen, Thermo Fisher Scientific) with a ×40 objective lens and a high-performance dual camera was used for imaging experiments. The microscope was automatically controlled and quickly clipped view profile by Micro-manager. NK cell cytotoxicity was assessed as previously described^38^. Captured images were processed and analyzed with ImageJ software.

**Subcutaneous xenograft mouse models for in vivo treatment**—All mouse experiments were performed in accordance with Guangzhou Medical University Experimental Animal Center and Institutional Animal Care and Use Committee (IACUC) guidelines and were approved by Guangzhou Medical University IACUC. All protocols were approved by the IACUC. All mice were maintained in specific pathogen-free (SPF)-grade cages and were provided autoclaved food and water in the Animal Core Facility of Guangzhou Medical University. Considering the increased cytokine production and activation capacity of CLDN6-CAR_1_ NK cells and the better antitumor activities, CLDN6-CAR_1_ NK cells were applied in the following in vivo antitumor assay.

For cell line-based ovarian cancer (OC), subcutaneous (s.c.) xenograft models to assess CDLN6-CAR_1_ NK cytotoxicity, 1 × 10^6^ SK-OV-3 GL cells in 100 μl PBS were injected subcutaneously into the right flanks of NSG mice aged 4-5 weeks on day 0. When tumor nodes were palpable (approximately 100 mm^3^), the mice were divided into three groups (NC, CD19-CAR NK, and CLDN6-CAR_1_ NK, n=5) and received 5 × 10^6^ irradiated CLDN6-CAR_1_ NK cells or CD19-CAR NK cells in 100 μl PBS intravenously on day 11. Mouse body weight was monitored regularly by electronic scales. Tumor volume was measured regularly with a caliper and calculated by the following equation: tumor volume (mm^3^) = (length × width^2^)/2. All mice were sacrificed on day 43. The mice were dissected, and their organs and tumor tissues were fixed in 4% paraformaldehyde fixative for H&E staining and immunohistochemistry.

To compare the efficacy of peritumoral and intravenous delivery of CAR-NK cell therapy, 1 ×10^6^ OVCAR-3 GL cells in 100 μl PBS were injected subcutaneously into the right flanks of NSG mice. Then, approximately ten days after tumor cell injection, the mice were subjected to BLI and randomly divided into five groups: NC, CD19-CAR NK i.v., CD19-CAR NK p.t., CLDN6-CAR_1_ NK i.v., CLDN6-CAR_1_ NK p.t., and injected with 5 ×10^6^ irradiated CLDN6-CAR_1_ NK cells or the same number of irradiated CD19-CAR NK cells in 100 μl PBS intravenously or peritumorally (every 7days, twice in total). On days 17 and 28, the mice were subjected to BLI analysis again. Euthanasia was performed according to Rutgers IACUC guidelines when tumor load >2000 mm^3^ or animal weight loss > 20%^20^. Cell suspensions were prepared from mouse spleens and subcutaneous tumors by tissue disruption with glass slides, filtering through a 40 μm filter, lysing with ACK lysis buffer (Biolegend) and digesting with Accutase (Gibco)^39^. For analysis of mouse peripheral blood, blood was collected by retroorbital bleeds into EDTA FACS tubes and underwent two rounds of lysis with ACK lysis buffer (Biolegend)^39^. Single-cell suspensions were washed and prepared for flow cytometry analysis as indicated at the second week after CAR-NK cells infusion. Tumor specimens from tumor-bearing mice were fixed in 10% neutral-buffered formalin and used for IHC analysis to detect CD3 expression.

For experiments analyzing the effect of anti-PD-L1 on CLDN6-CAR_1_ NK antitumor activity and persistence, 1 × 10^6^ PD-L1+ Hey GL cells in 100 μl PBS were injected subcutaneously into the right flanks of NSG mice. Then, approximately nine days after cancer cell injection, the mice were subjected to BLI and randomly divided into four groups, NC, anti-PD-L1, CLDN6-CAR_1_ NK, and CLDN6-CAR_1_ NK + anti-PD-L1, and peritumorally injected with 5 × 10^6^ irradiated CLDN6-CAR_1_ NK cells with or without 10mg/kg anti-PD-L1 antibody on days 10 and 16 or anti-PD-L1 and PBS alone. Then, 6 days later, the mice that used anti-PD-L1 were injected with the same amount of anti-PD-L1 again. On days 15 and 27, the mice were subjected to BLI again. Tumor specimens from tumor-bearing mice were fixed in 10% neutral-buffered formalin and used for IHC analysis to detect CD3 expression. Single-cell suspensions from mouse blood, tumors and spleens were washed and prepared for flow cytometry analysis following previously described methods.

**Intraperitoneal xenograft mouse models for in vivo treatment**—For the intraperitoneal OC xenograft model, the human OC cell line SK-OV-3 GL (1 × 10^6^ cells/mouse) suspended in 100 μl PBS was inoculated intraperitoneally (i.p.) into 4-5-week-old female NSG mice. Thirteen days after tumor inoculation, the mice were subjected to BLI and randomly divided into three groups: NC, CD19-CAR NK, and CLDN6-CAR_1_ NK groups. Mice received irradiated CLDN6-CAR_1_ NK cells (5 × 10^6^ cells/mouse) or an equivalent number of irradiated CD19-CAR NK cells suspended in 100 μl PBS intraperitoneally on days 14 and 21. On days 20 and 34, the mice were subjected to BLI again. For analysis of mouse peripheral blood, blood was collected by tail vein bleeds into EDTA FACS tubes and underwent two rounds of lysis with ACK lysis buffer (Biolegend) at the second week after CAR-NK cell infusion. The single-cell suspension was washed and prepared for flow cytometry analysis as indicated.

To further analyze the effect of anti-PD-L1 on CLDN6-CAR_1_ NK antitumor activity and persistence, 1 × 10^6^ PD-L1+ Hey GL cells in 100 μl PBS were inoculated intraperitoneally into NSG mice. Fourteen days after tumor inoculation, the mice were subjected to BLI and randomly divided into four groups: NC, anti-PD-L1, CLDN6-CAR_1_ NK, and CLDN6-CAR_1_ NK+anti-PD-L1 groups. Mice were intraperitoneally injected with 5 × 10^6^ irradiated CLDN6-CAR_1_ NK cells with or without 10mg/kg anti-PD-L1 antibody on days 15 and 22 or treated with anti-PD-L1 or PBS. Then,7 days later, the mice that used anti-PD-L1 were injected with the same amount of anti-PD-L1 again. On days 21, 26 and 36, the mice were subjected to BLI again. A single-cell suspension from mouse blood was washed and prepared for flow cytometry analysis following previously described methods. Mice were euthanized when signs of discomfort or 20% weight loss were detected by the investigators who monitored the mice three times a week.

**Mouse systemic tumor models for in vivo treatment**—For the mouse OC metastatic model, 4- to 5-week-old female mice were inoculated with 1 × 10^6^ OVCAR-3 GL cells by tail vein injection. The mice were subjected to BLI on day 16 and divided into three groups: NC, CD19-CAR NK, and CLDN6-CAR_1_ NK groups. Seventeen days after tumor inoculation, 1 to 5 × 10^6^ irradiated CLDN6-CAR_1_ NK or CD19-CAR NK cells were injected intravenously on days 17 and 25, respectively. On days 24 and 36, the mice were subjected to BLI again. Mice were matched based on the tumor bioluminescence before assignment to control or treatment groups. Mice were euthanized when signs of discomfort or 20% weight loss were detected. Then, the lung tissue of mice was collected, and the number of nodules on the lung surface was counted. For all NSG mouse experiments, CAR-NK cells were FACS sorted to >99% purity and expanded for 4-5 days prior to injection.

**Bioluminescence imaging (BLI)**—All tumor burdens were monitored by IVIS bioluminescence imaging. For bioluminescence imaging of luciferase-labeled tumor cell growth in vivo, mice received intraperitoneal injections of D-Luciferin substrate (sodium salt; Yeasen BioTechnology) resuspended in PBS (150 µg/g body weight). Mice were anesthetized with isoflurane and imaged 10-15 min after D-luciferin was injected with a PerkinElmer IVIS Imaging System (PerkinElmer, USA) at the indicated time points as previously described^39^. Quantification of total and average emissions was performed with Living Image version 4.7.3 software (PerkinElmer).

### Statistical analysis

All data are presented as the mean values ± SD or SEM unless indicated otherwise. Two-tailed unpaired t-test, one-way ANOVA, and two-way ANOVA were used. Bonferroni's correction for multiple comparisons was used to calculate the adjusted p value when appropriate. For survival analysis, Kaplan-Meier curves were plotted and compared by the log-rank test. Statistical analyses were performed using GraphPad Prism software 8.0. The exact p values are shown in the figures; ns, not significant. Statistical significance was established at the levels of *, p<0.05; **, p<0.01; ***, p<0.001; ****, p<0.0001.

## Results

### Expression profile of CLDN6 in ovarian cancer tissues, cell lines and normal human tissues

**Figure 1:**
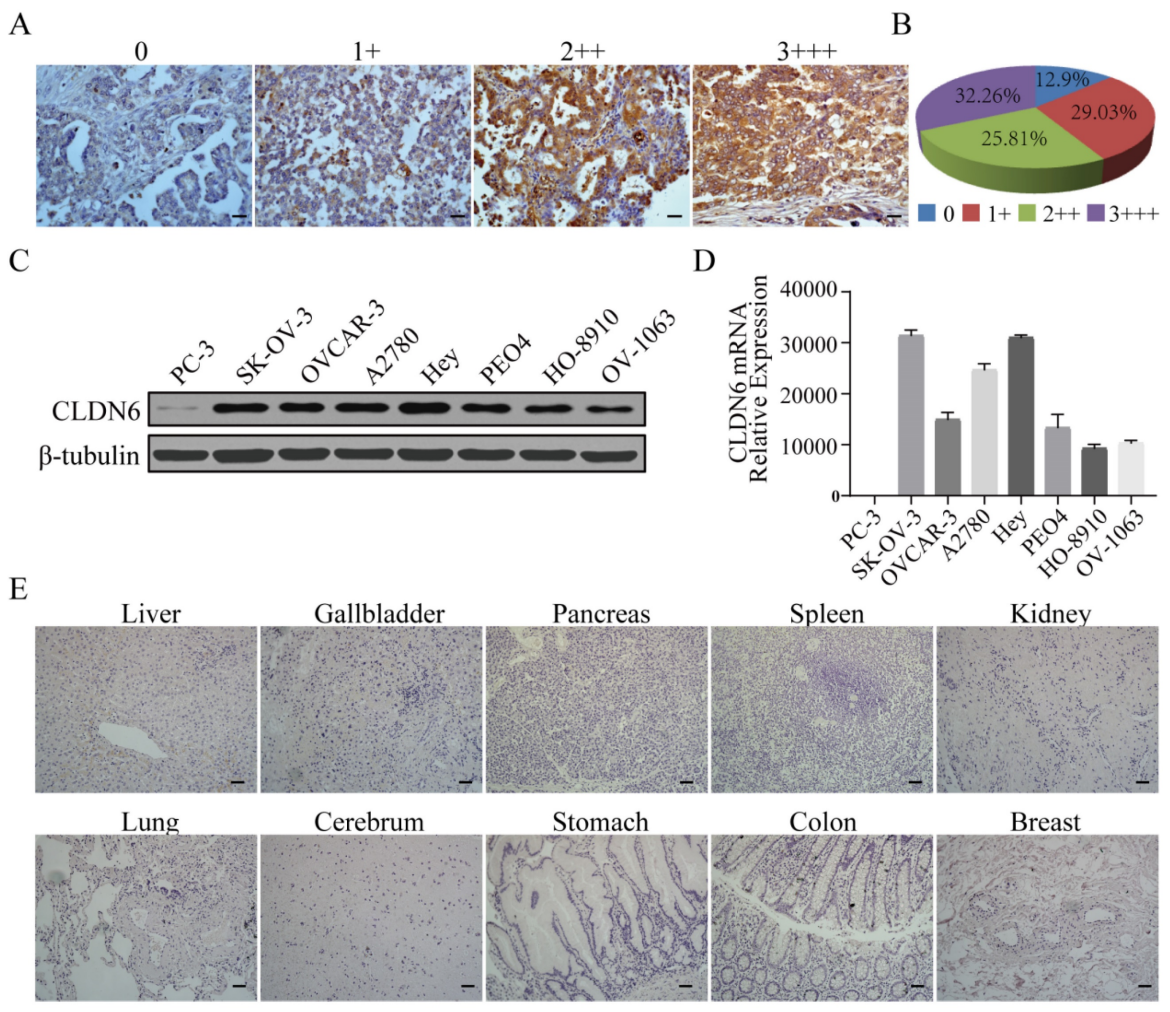
** Analysis of CLDN6 expression in human normal tissue, primary ovarian cancer tissues, and human ovarian cancer cells.** (A) Different levels of CLDN6 expression in primary ovarian cancer tissues were evaluated by two experienced pathologists using a 4-point scale at 400× magnification; scale bar, 50 µm. (B) The percentage of CLDN6-positive staining with different scores in 62 primary ovarian cancer samples is indicated. (C) Expression of CLDN6 in human ovarian cancer cell lines assessed by western blot with the anti-CLDN6 mAb. (D) Relative expression of CLDN6mRNA normalized to GAPDH in various human ovarian cancer cell lines was assessed by qPCR. (E) Ten human normal tissue samples were immunostained with an anti-CLDN6 antibody to determine the expression of CLDN6 at 200× magnification; scale bar, 100 µm.

Tumor targeting by CAR-NK cells requires the expression of certain tumor-associated antigens (TAAs) on the surface of tumor cells. We performed immunohistochemical staining for CLDN6 in 62 cases of primary OC samples and found that 87.1% (54/62) of ovarian cancer tissues showed positive expression of CLDN6 and localized to the cell membrane. Among them, 32.3% (20/62) showed strong positive expression (Fig. 1A, 1B). We examined CLDN6 expression in seven human OC cell lines, OVCAR-3, SK-OV-3, A2780, Hey, PEO4, HO-8910 and OV-1063 by Western blotting (WB). All seven OC cell lines expressed CLDN6, but PC-3 cells had a low expression (Fig. 1C). Therefore, PC-3 was used as a negative control in our study. RT-PCR analysis showed that CLDN6 mRNA had different transcriptional levels in seven human ovarian cancer cell lines, and the expression levels of SK-OV-3, A2780 and Hey were higher (Fig. 1D). In addition, CLDN6 expression was not detected in normal tissues of 11 important human organs (Fig. 1E). Collectively, these results indicate that CLDN6 expression is upregulated in both OC primary tissues and cell lines and CLDN6 can be used as an ideal target for ovarian cancer treatment.

### Generation of CLDN6-specific CAR-modified NK-92MI cells

According to the characteristics of NK cells' self-activated receptors and related reports^15^, we plan to utilize the activation elements (NKG2D, 2B4) of NK cells to construct the CAR molecular structure of transmembrane and intracellular costimulatory signals of CAR molecules (CAR_1_) and to explore whether it can recognize the transmission and enhancement of CAR signals and whether it is superior to the classical CAR-T cell activation element structure (CAR_2_: including CD28 and 4-1BB).

Among them, NKG2D is an important activated receptor in NK cells, 2B4 is an important signal regulatory molecule in NK cells, DNAX-activating protein 10 (DAP10) plays an important role in intracellular signal transduction in NK cells, and DAP10 in tandem with CD3ζ serves as the intracellular signal transduction domain in NK cells. Therefore, we constructed two CAR molecular lentiviral vectors targeting CLDN6 for this experiment (CLDN6-CAR_1_ and CLDN6-CAR_2_) and constructed the CAR molecular structure targeting CD19 as the control (CD19-CAR) in the experiment (Fig. S1A). The three CAR lentiviral vectors were successfully constructed and confirmed by agarose gel electrophoresis (Fig. S2A). HEK-293T packaged lentivirus and lentivirus titers were detected (Table S1). After CAR lentivirus transfection, enrichment and purification, the transduction efficiency was determined by the percentage of green fluorescent protein-positive (GFP+) cells detected by flow cytometry. CLDN6-CAR_1_, CLDN6-CAR_2_ and CD19-CAR molecules were highly purified in CAR-NK cells, with GFP reporter 99.5%, 99.6% and 99.9%, respectively (Fig. S1B and Fig. S2B). We further confirmed that the CAR sequences were introduced and expressed in NK-92MI cells by agarose gel electrophoresis, qPCR and WB (Fig. S1C, S1D, S1E). Morphological identification of NK cells was also carried out by confocal microscopy. The size and shape of NK cells in the resting state were good, and GFP was expressed in each cell (Fig. S3). These results confirmed that CAR-NK cells were generated successfully. CD19-CAR NK cells containing classical domains (CD28 and 4-1BB) have been verified in previous experiments^37^, so they will not be repeated in subsequent experiments.

### CLDN6-CAR NK-92MI cells showed strong antitumor activity against ovarian cancer cell lines in vitro

**Figure 2:**
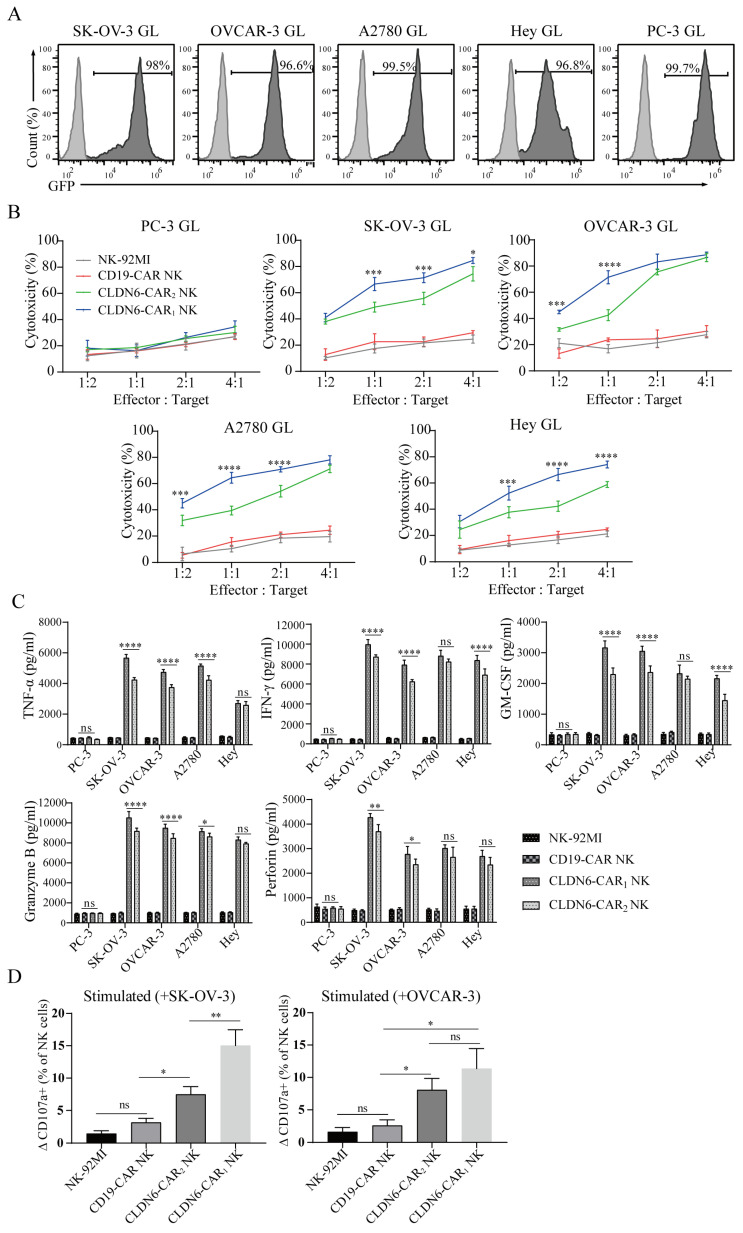
** Cytotoxicity activities and cytokine secretion of CLDN6-specific NK-92MI cells in vitro.** (A) Luciferase and GFP (GL) expression detected in the indicated genetically modified human ovarian cancer cells by FACS. GFP was used as a detection marker. (B) The indicated genetically modified and parental NK cells were coincubated with different target cells at varying effector to target (E:T) ratios for 8 h. Cell lysis was tested using a standard nonradioactive cytotoxicity assay. The graphed results are presented as the mean ± SD of three or more independent experiments. Error bars denote SD. ***p < 0.001, ****p < 0.0001, two-tailed Student's t test adjusted p value. (C) The production of TNF-α, IFN-γ, GM-CSF, granzyme B, and perforin by CAR-NK cells after coculture with target cells for 24 h at a 1:1 E:T ratio was determined by enzyme-linked immunosorbent assay (ELISA). CLDN6-CAR_1_ NK and CLDN6-CAR_2_ NK are statistically compared with NK-92MI and CD19-CAR NK samples (p < 0.0001). Error bars denote SD. ns, not significant, * p < 0.05, **p < 0.01, ****p < 0.0001, two-tailed Student's t test adjusted p value. (D) Detection of the cell surface activation marker CD107a on CAR-NK cells after stimulation with SK-OV-3 and OVCAR-3 cells at an E:T ratio of 1:1 or no stimulation. We defined the y-axis as the ΔCD107a+ percentage of NK cells minus the CD107a+ percentage in resting NK cells from the CD107a+ percentage of NK cells stimulated with target cells. Error bars denote SD. ns, not significant, *p < 0.05, **p < 0.01, one-way ANOVA with Holm-Sidak test adjusted p value. See also Figure S4.

To determine the cytotoxicity of CAR-NK cells against ovarian cancer cell lines in vitro, an LV5-LUC-GFP-Puro transgene (GL) was lentivirally transduced into 5 cancer cell models which the SK-OV-3 GL, OVCAR3 GL, A2780 GL, Hey GL and PC-3 GL cell lines were constructed (Fig. 2A). To enrich GL cells expressing GFP markers, two or three rounds of cell sorting using a FACS sorter were carried out. Flow cytometry results indicated that luciferases were expressed and highly purified in GL cells with the GFP reporter (98.0%, 96.6%, 99.5%, 96.8% and 99.7%, respectively) (Fig. 2A). We then performed an 8-h killing assay of NK-92MI, CD19-CAR, CLDN6-CAR_1_ and CLDN6-CAR_2_ NK cells on the 4 ovarian cancer GL-expressing cell lines. The results showed that both CLDN6-CAR_1_ and -CAR_2_ NK cells exhibited stronger cytotoxicity than CD19-CAR NK or parental NK-92MI cells after coculture with all 4 ovarian cancer cell lines at the indicated effector-to-target (E:T) ratios but not CLDN6-negative PC-3 GL cells (Fig. 2B). Notably, CLDN6-CAR_1_ NK cells had higher cytotoxic activities against OC cells than CLDN6-CAR_2_ cells, especially at E:T = 1:1 or 1:2 ratios, in vitro (Fig. 2B). Cytokine secretion of the CLDN6-CAR_1_ NK and CLDN6-CAR_2_ NK cells after target cell stimulation was further evaluated by enzyme-linked immune absorbance assay (ELISA). As shown in Figure 2C, a significant increase in tumor necrosis factor α (TNF-α), interferon-γ (IFN-γ), granulocyte-macrophage colony stimulating factor (GM-CSF), perforin and granzyme B was produced by two CLDN6-CAR NK cells compared with CD19-CAR NK or parental NK-92MI cells in the presence of SK-OV-3 GL, OVCAR-3 GL, A2780 GL and Hey GL (CLDN6 positive), whereas this phenomenon was not observed in the presence of PC-3 GL (CLDN6 negative) (Fig. 2C). Overall, CLDN6-CAR_1_ NK cells possessed stronger cytokine-secreting capabilities than CLDN6-CAR_2_ NK cells (Fig. 2C).

In addition, CD107a expression positively correlates with NK cell degranulation, a key process responsible for cytotoxicity against target cells. We performed flow cytometry study to test the levels of CD107a on CAR-NK cells after stimulation with SK-OV-3 or OVCAR-3 target tumor cells. The results showed that CD107a on CLDN6-CAR_1_ and CLDN6-CAR_2_ NK cells was upregulated more obviously after stimulation with target cells compared with unstimulated cells, CD19-CAR NK cells and parental NK-92MI cells (Fig. 2D and Fig. S4). Interestingly, the level of CD107a on CLDN6-CAR_1_ NK cells after stimulation with SK-OV-3 was higher than that on CLDN6-CAR_2_ NK cells, while there was no difference with OVCAR-3 cells (Fig. 2D and Fig. S4). In conclusion, these data indicated that in contrast to parental NK-92MI or CD19-CAR NK cells, both CLDN6-CAR NK cells exhibited robust cytotoxicity and cytokine production as well as NK cell activation after they encountered target CLDN6-positive tumor cells in vitro, which suggests that CLDN6-CAR NK cells can elicit strong immune responses and antitumor activity against ovarian cancer cells with CLDN6 expression. Intriguingly, CLDN6-CAR_1_ NK cells constructed with their own activation element had a better killing effect than CLDN6-CAR_2_ NK cells constructed with the classical CAR structure.

### Evaluation of CAR-NK92MI cell killing efficacy in vitro based on live-cell dynamic imaging

**Figure 3:**
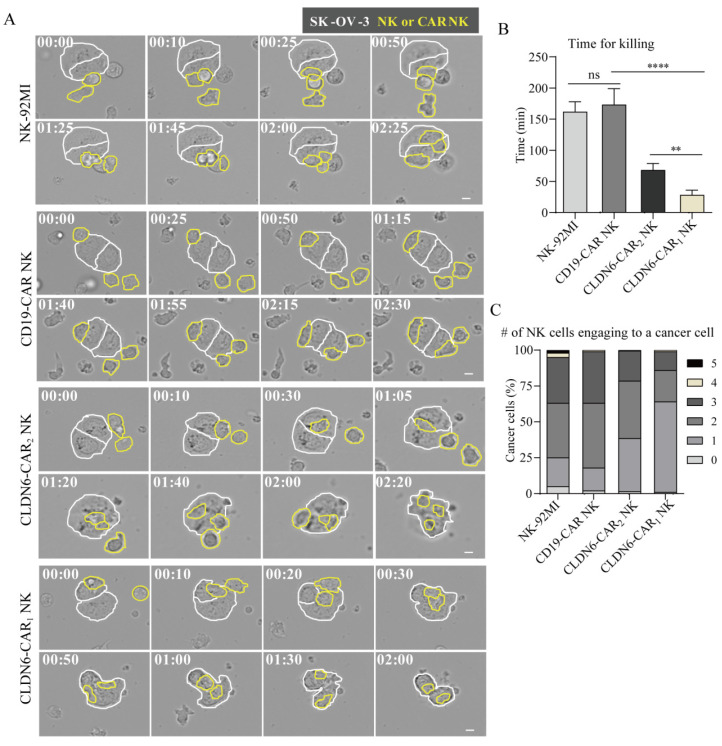
** Analysis of CAR-NK92MI cell cytotoxicity based on live cell imaging for SK-OV-3 cells.** (A) Representative time-lapse images of the interaction between different CAR-NK cells (yellow lines) and SK-OV-3cells (white lines). (B-C) Time for killing (B) and the number of NK cells engaging with a cancer cell (C). Error bars denote SD. ns, not significant, **p < 0.01, ****P<0.0001, Mann-Whitney test adjusted p value. See also Figure S5 and S6 and online supplementary videos S1-4. A time display on the left upper corner indicated in (A) represents the changed time post CAR-NK cell coculture with target cells.

Standard cytotoxicity analyses usually provide population-level information and do not provide any information on functional heterogeneity at the single-cell level, whereas cytotoxicity analyses based on dynamic live-cell imaging can assess heterogeneity and more details at the single-cell level. Under the microscope, NK cells show morphological changes and dynamic behaviors of exploring the extracellular space through membrane extension^40^. We used a live cell imaging system to evaluate the killing process of NK cells to target cells at the single-cell level, directly observe the interaction patterns between different NK cells and target cells, and quantitatively evaluate cytotoxicity at the single-cell level. NK-92MI and CAR-NK cells were added to SK-OV-3 cells and observed by time-lapse imaging (Fig. 3A and online supplementary videos S1-4). SK-OV-3 cells (marked with white boundaries in Figure 3A) and NK-92MI or CAR-NK cells (marked with yellow boundaries in Figure 3A) showed distinct behaviors: SK-OV-3 cells were uniformly attached to the bottom of the dish and barely moved, while NK cells migrated actively by chasing and attaching to SK-OV-3 cells. The membrane of SK-OV-3 cells contacted by NK cells showed bubbles, abnormal morphology, disappeared outline, detached from the substrates and became round and cracked at the end, indicating that SK-OV-3 cells were destroyed by the CAR NK cells^41^, ^42^. Only a fraction of NK-92MI and CD19-CAR NK cells exerted cytotoxicity, while the majority of CLDN6-CAR NK cells contacting cancer cells showed obvious killing behavior, with thin lamellar protrusions and filar structures formed in the cell front.

Of note, compared with CLDN6-CAR NK cells, NK-92MI and CD19-CAR NK cells contacting cancer cells also underwent morphological changes, namely, short membrane extension, flap-shaped membrane extension or vesicular protrusion, and partial morphology elongation, indicating that natural biological characteristics of NK cells still exist, but they lacked the ability to specifically recognize antigens and showed little cytotoxicity.

To further assess cytotoxicity at the single-cell level, we measured the killing time for each NK cell against cancer cells, defined as the duration between NK cell engagement with cancer cells and the onset of cancer cell blebbing (Fig. 3B). The killing time of the NK-92MI group was similar to that of the CD19-CAR-NK group, which was ~ 170 min, while that of the CLDN6-CAR_2_ NK group was <70 min and that of the CLDN6-CAR_1_ NK group was <30 min. There was a significant difference among the killing times (Fig. 3B). Compared with the control group, the killing time of NK cells in the CLDN6-CAR_2_ NK group was significantly shortened by more than 2 times, and the killing time of NK cells in the CLDN6-CAR_1_ NK group was shortened by more than 5 times, further indicating that self-activating elements (NKG2D, 2B4) as the CAR domain improved the killing efficiency of CAR_1_-NK cells targeting CLDN6. These results were consistent well with the assays of luciferase to detect cell killing ability.

The killing effect of NK cells on cancer cells not only requires that NK cells have strong cytotoxicity but also depends on a certain number of NK cells to complete the killing ability. The accelerated killing of tumor cells mediated by the number of NK cells often leads to the attachment of more NK cells around the tumor cells; that is, the death of a cancer cell requires the participation of multiple NK cells. To evaluate the number of NK cells needed to kill a cancer cell, the number of NK cells attached to each cancer cell (Fig. 3C) was measured. In the NK-92MI group and the CD19-CAR NK group, more than 70% of the cancer cells were surrounded by 2-3 NK cells, and the cytotoxicity was extremely limited. In contrast, most cancer cells in the CLDN6-CAR_2_ NK and CLDN6-CAR_1_ NK groups were surrounded by 1 or 2 NK cells. In the CLDN6-CAR_1_ NK group, one NK cell was enough to kill one cancer cell without the need for additional NK cells (Fig. 3C). In addition, CLDN6-CAR_1_ NK cells also had a certain continuous killing capability; that is, after killing one cancer cell, they could migrate and kill the second cancer cell (Fig. S5), which was sufficient to demonstrate its strong cytotoxicity and lasting killing activity.

For PC-3 cancer cells that did not express CLDN6, NK-92MI, CD19-CAR NK, CLDN6-CAR_1_ NK and CLDN6-CAR_2_ NK cells showed static round shapes and minimal membrane extension. With the passage of time, some cells also changed into elongated shapes, and a few filaments appeared in the membrane extension. However, no significant cytotoxicity was shown due to a lack of specific antigen activation (Fig. S6A). The interaction time between NK cells and cancer cells was significantly prolonged, and the killing ability was very limited. Meanwhile, the number of NK cells required was also relatively increased (Fig. S6B, S6C). Altogether, a single-cell level cytotoxicity assay based on live cell imaging revealed that the powerful CAR_1_-NK cells targeting CLDN6 constructed by self-active elements significantly enhanced the specifical killing efficacy against CLDN6-positive cancer cells.

### Compared to intravenous delivery, peritumoral delivery of the CLDN6-CAR NK92MI cells significantly improved their tumor infiltration and cancer cell killing efficacy in vivo

**Figure 4:**
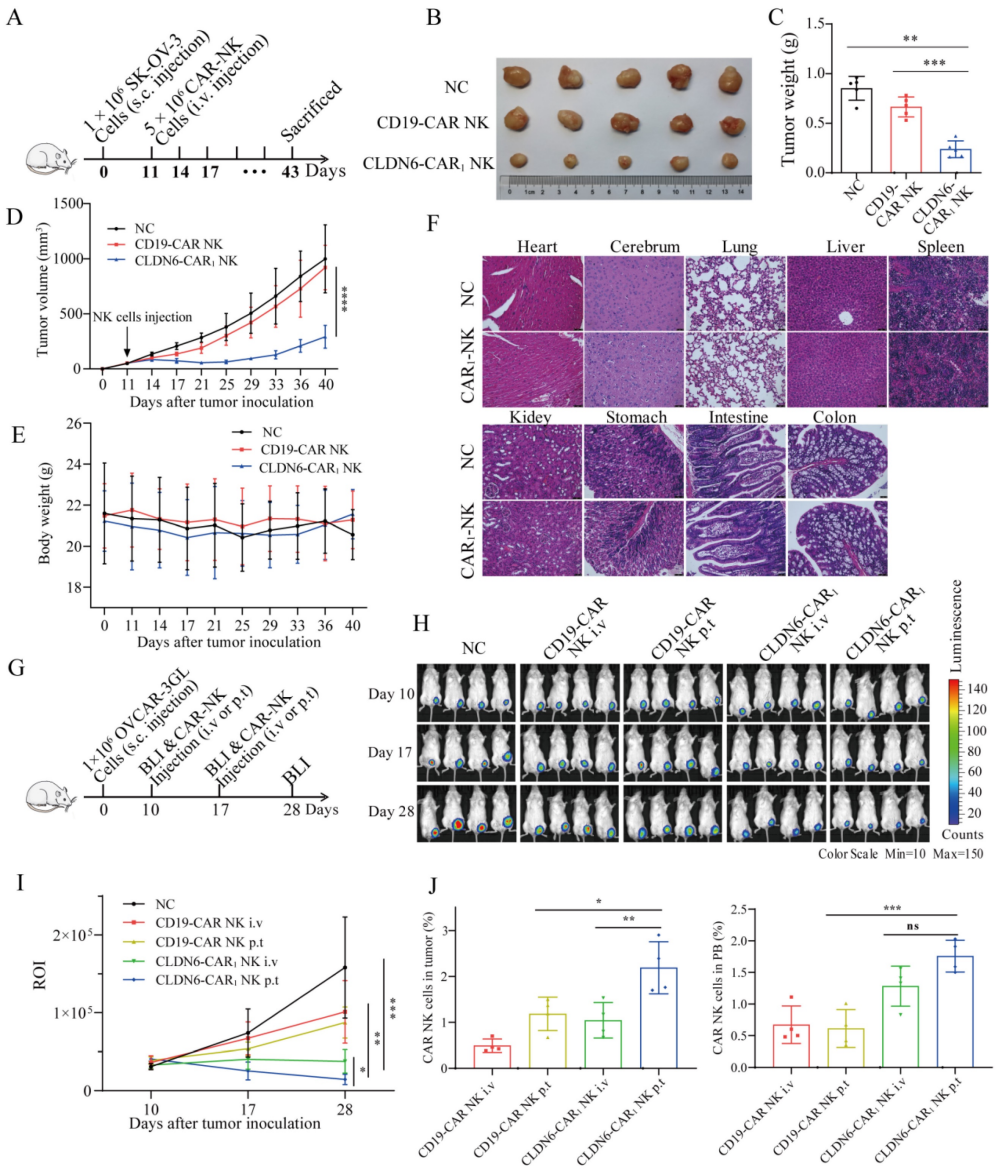
** CLDN6-CAR_1_ NK92MI cells suppress tumor growth in NSG mice without causing evident toxicity.** (A) Schematic representation of the s.c. xenograft model experiments. NSG mice received 1 × 10^6^ SK-OV-3 cells with subcutaneous injection; 5 × 10^6^ CAR-NK cells were administered through the tail vein on day 11, and tumor volume and mouse body weight were regularly measured. (B-E) Tumors dissected from different groups at the end point (B), tumor weight in each group (C), tumor volume curves (D), and mouse body weight (E) (5 mice/group). Error bars denote SD. **p < 0.01, ***p < 0.001, ****p < 0.0001, one-way ANOVA with Holm-Sidak test adjusted p value. (F) Histopathological analysis of mouse organ tissues by hematoxylin and eosin (H&E) staining. Representative photomicrographs are shown (magnification ×20). Each scale bar represents 100 µm. (G) Schematic representation of the experiments. NSG mice received 1 × 10^6^ OVCAR-3 GL cells with subcutaneous (s.c.) injection; 5 × 10^6^ CAR-NK cells were administered intravenously (i.v.) or peritumorally (p.t.) on days 10 and 17, and BLI was performed regularly. (H and I) Representative bioluminescence images (H) and bioluminescence kinetics ROI (I) of OVCAR-3 GL tumor growth in the model shown in (G) (4 mice/group). (J) Mice were euthanized 12-14 days post CAR-NK cell infusion to analyze the infiltration percentages of CAR-NK cells in tumors and PB by flow cytometry. Error bars denote SD. *p < 0.05, **p < 0.01, ***p < 0.001, one-way ANOVA with Holm-Sidak test adjusted p value.

Considering the increased cytokine production and activation capacity of CLDN6-CAR_1_ NK cells and the better antitumor activities, CLDN6-CAR_1_ NK cells were applied in the following in vivo antitumor assay. Mouse subcutaneous (s.c.) OC model was inoculated with SK-OV-3 cells on day 0 and intravenously administrated with CLDN6- or CD19-CAR NK cells on day 11 (Fig. 4A). As shown in Fig. 4B and 4D, in the CLDN6-CAR_1_ NK groups, the calculated tumor volume remained relatively stable and low level, whereas in the NC and CD19-CAR NK groups, the tumor volume progressed obviously. Accordingly, the tumor weight in the CLDN6-CAR_1_ NK groups was lower than that in mice in the NC and CD19-CAR NK groups (Fig. 4C). Moreover, no obvious damage was observed in the important organs from the mice treated with CLDN6-CAR_1_ NK cells (Fig. 4F). The weight of mice treated with CAR_1_-NK cells did not change obviously (Fig. 4E). All these data support that CLDN6-CAR_1_ NK cells did not induce on-target off-tumor toxicities in the in vivo mouse models.

To further optimize and improve the therapeutic effect in vivo, we explored different NK cell administration approaches^20^, ^43^. OVCAR-3 GL cells were subcutaneously transplanted into NSG mice. After 10 days, the mice were subjected to bioluminescence imaging (BLI) to confirm that subcutaneous tumor model was successfully constructed. Then, mice were treated with CD19-CAR NK cells or CLDN6-CAR_1_ NK cells via different delivery methods, including intravenous (i.v.) or peritumoral (p.t.) injection on days 10 and 17 (Fig. 4G). The mice were divided into five groups: blank, CD19-CAR NK i.v., CD19-CAR NK p.t., CLDN6-CAR_1_ NK i.v., and CLDN6-CAR_1_ NK p.t (Fig. 4H). In total, 5 × 10^6^ CLDN6-CAR_1_ NK cells were injected into the mice in each of the CLDN6-CAR_1_ NK groups, and the same number of CD19-CAR NK cells was injected into the mice in the CD19-CAR NK cell group (Fig. 4G and 4H). P.t. delivery of CAR-NK cells inhibited the growth of subcutaneous tumors more significantly in comparison to i.v. delivery, as detected by BLI on days 17 and 28 (Fig. 4H and 4I). Persistence of CAR-NK cells was detected in peripheral blood (PB) and tumors at the second week after CAR-NK cell infusion (Fig. 4J). More importantly, NK cell infiltration in the CLDN6-CAR_1_ NK p.t. group was higher than that in the CLDN6-CAR_1_ NK i.v. group as detected by flow cytometry, which further warranted the therapeutic effects of CLDN6-CAR_1_ NK cells to treat ovarian cancer (Fig. 4J). Collectively, these results demonstrated that CLDN6-CAR_1_ NK cells could suppress ovarian cancer progression in the s.c. mouse models and the peritumoral delivery approach significantly enhanced CAR-NK cell infiltration into solid tumors compared with intravenous delivery.

### CLDN6-CAR NK92MI cells showed strong antitumor activity against OC in vivo in both intraperitoneal and hematogenic metastasis models

**Figure 5:**
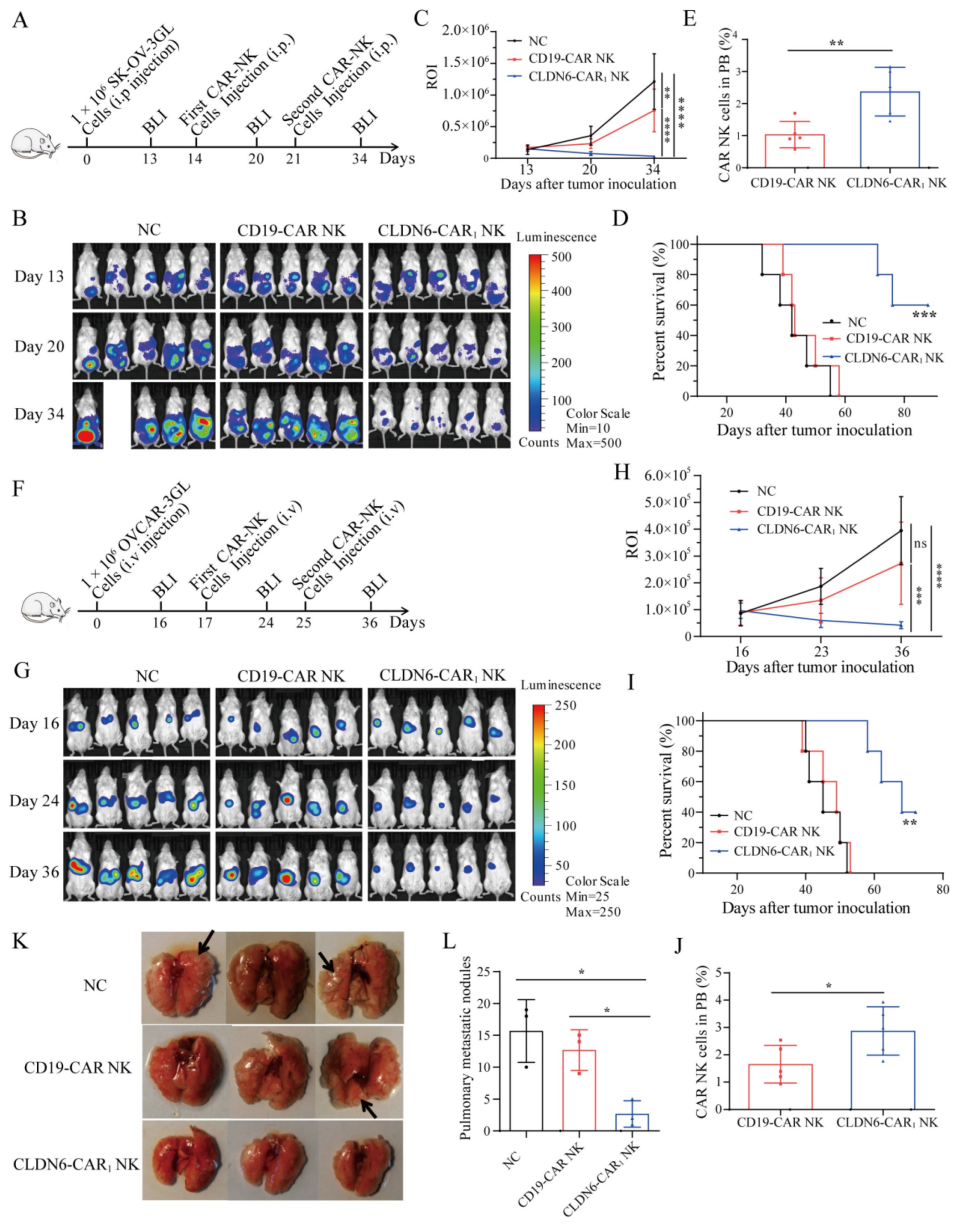
** CLDN6-CAR_1_ NK92MI cells showed strong antitumor activity in vivo in both intraperitoneal and systemic metastatic OC models.** (A) Schematic representation of the experiments. NSG mice received 1 × 10^6^ SK-OV-3 GL cells intraperitoneally; 5 × 10^6^ CAR-NK cells were administered intraperitoneally on days 14 and 21, and BLI was performed on days 13, 20 and 34. (B and C) Representative bioluminescence images (B) and bioluminescence kinetics ROI (C) of SK-OV-3 GL tumor growth in the model shown in (A) (5 mice/group). Error bars denote the SD. **p < 0.01, ****p < 0.0001, one-way ANOVA adjusted p value. (D) Kaplan-Meier survival curve of SK-OV-3 GL cells intraperitoneally injected into mice (5 mice/group). ***p = 0.0006, log-rank test. (E) Twelve to 14 days after CAR-NK cell infusion, the infiltration percentages of CAR-NK cells in PB were analyzed by flow cytometry. Error bars denote SD. **p < 0.01, two-tailed Student's t test adjusted p value. (F) Schematic representation of the mouse OC systemic metastatic model experiments. NSG mice received 1 × 10^6^ OVCAR-3 GL cells via the tail vein; 5 × 10^6^ CAR-NK cells were administered intraperitoneally on days 17 and 25, and BLI was performed on days 16, 24 and 36. (G and H) Representative bioluminescence images (G) and bioluminescence kinetics (H) of OVCAR-3 GL tumor growth in the model shown in (F) (5 mice/group). Error bars denote the SD. ns, not significant, *** p < 0.001, **** p < 0.0001, one-way ANOVA adjusted p value. (I) Kaplan-Meier survival curve of OVCAR-3 GL cells intravenously injected mice (5 mice/group). ** p = 0.0011, log-rank test. (J) Fourteen days after CAR-NK cell infusion, the infiltration percentages of CAR-NK cells in PB were analyzed by flow cytometry. Error bars denote SD. *p < 0.05, two-tailed Student's t test adjusted p value. (K and L) Lung dissected from different groups (K) and the number of nodules (blank arrow) on the lung surface at the end point (L) (3 mice/group). Error bars denote SD. *p < 0.05, one-way ANOVA adjusted p value.

The main modes of ovarian cancer metastasis are local diffusion metastasis and peritoneal implantation, while peritoneal administration of chemotherapy is the main treatment for ovarian cancer^44^. To further verify the efficacy of CLDN6-CAR_1_ NK cells against CLDN6-positive OC cells in vivo, we established several different human OC xenograft mouse models. First, the mice were injected with SK-OV-3 GL cells in peritoneal cavity on day 0 to construct an intraperitoneal ovarian cancer model. On day 13, the mice were subjected to BLI and robust intraperitoneal expansion of tumor cells was observed (Fig. 5A). These mice were then divided into three groups: the blank group, CD19-CAR NK cell group, and CLDN6-CAR_1_ NK cell group (Fig. 5A). Intraperitoneal infused CLDN6-CAR_1_ NK cells induced significant regression of SK-OV-3 GL OC cells, while tumors in the blank and CD19-CAR NK groups continued to progress, as detected by BLI on days 20 and 34 (Fig. 5B and 5C). Moreover, the majority of mice treated with CLDN6-CAR_1_ NK cells survived longer, whereas the median survival of mice in the blank and CD19-CAR NK groups was only 42 and 45 days, respectively, which was significantly shorter than that in the CLDN6-CAR_1_ NK group (Fig. 5D). The persistence of CAR-NK cells was detected in the PB at the second week after CAR-NK cell infusion (Fig. 5E). Taken together, these results suggested that CLDN6-CAR_1_ NK cells could effectively eradicate intraperitoneal ovarian cancer cells and prolong the survival time of tumor-bearing mice.

Advanced ovarian cancer can also metastasize through blood^45^, ^46^. To determine whether CLDN6-CAR_1_ NK cells can suppress the hematogenous metastasis of OC, we constructed another mouse model in which OVCAR-3 GL cells were intravenously injected into NSG mice (Fig. 5F). In the model, tumor cells were detected in the hepatic region (Fig. 5G), thus mimicking the hepatic metastasis of OC. After the same effector cells were injected into the tail vein, BLI results demonstrated that CLDN6-CAR_1_ NK cells almost inhibited tumor cell growth or even partially eliminated hepatic tumor cells in most of the mice on day 36, while NC and CD19-CAR NK cells could not control tumor cell progression (Fig. 5G and 5H). More importantly, we found that CLDN6-CAR_1_ NK cell therapy prolonged the survival time of the mice (Fig. 5I). A higher percentage of CAR-NK cells was detected in the CLDN6-CAR_1_ NK group at the second week after NK cell infusion (Fig. 5J). Lastly the tumor-bearing mice were euthanized according to the Institutional Animal Care and Use Committee (IACUC) guidelines and we dissected the mice and checked their lung surface nodules. The lung tissues of mice in the blank group and CD19-CAR NK group were covered with metastatic lung nodules of different sizes, most of which were bright and cystic, consistent with the cystic characteristics of ovarian cancer metastasis, while the lung nodules in the CLDN6-CAR_1_ NK group were scattered and few in number (Fig. 5K and 5L). The total lung tissue volume was also smaller than that in the other two groups (Fig. 5K). These findings demonstrated that CLDN6-CAR_1_ NK cells also exhibited strong antitumor activity in vivo against distant metastatic ovarian cancers.

### Contribution of CLDN6-CAR NK92MI cells to immunotherapy mediated by PD-1/PD-L1 blockade

**Figure 6:**
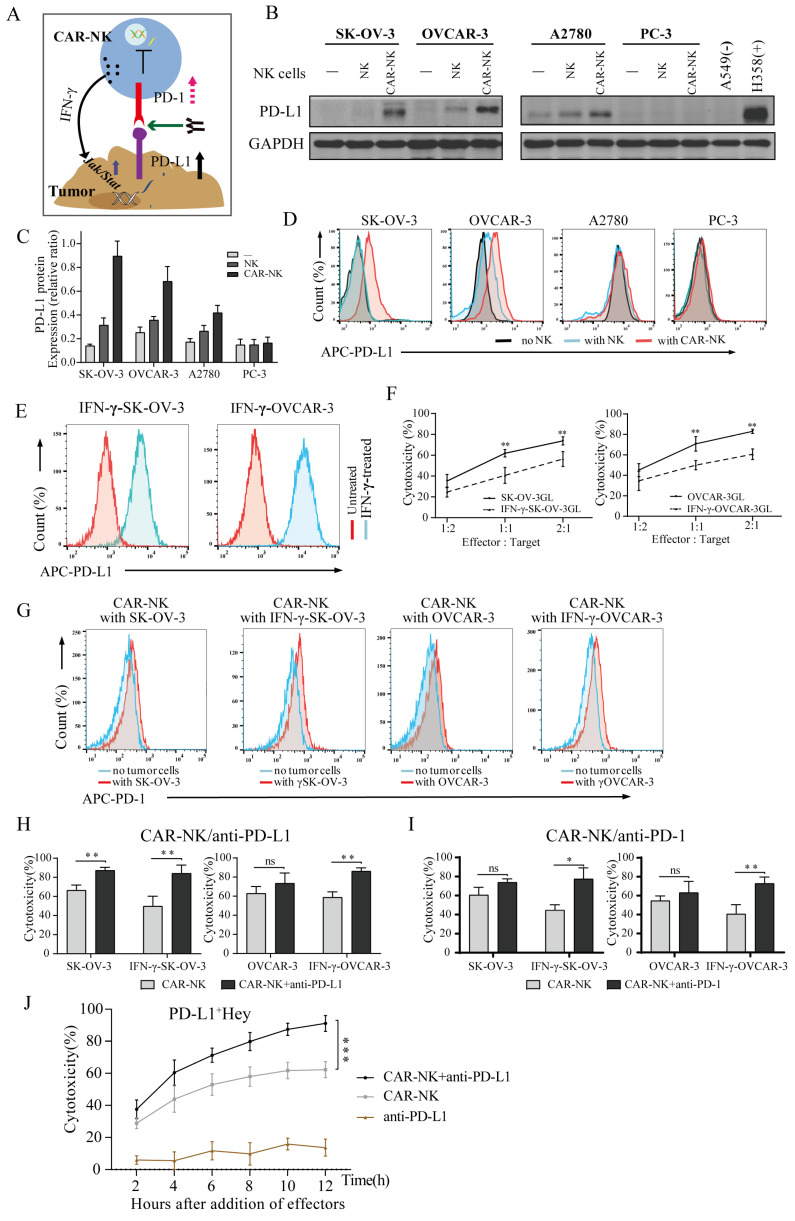
** Contribution of CLDN6-CAR_1_ NK92MI cells to immunotherapy mediated by PD-1/PD-L1 blockade.** (A) A cartoon depicting the tumor cell and CAR-NK cell interaction through the PD-1/PD-L1 axis. (B-D) The PD-L1 expression level of target cells after CAR-NK cells were cocultured with target cells (SK-OV-3, OVCAR-3, A2780 and PC-3) 6 hours was detected by western blot (B) with the anti-PD-L1 mAb and flow cytometry (D) with anti-PD-L1 conjugated with APC fluorochromes. A549 cells were used as a negative control, and H358 cells were used as a positive control. Relative expression of CLDN6 normalized to GAPDH was analyzed by ImageJ software (C). Error bars denote SD. (E) The PD-L1 expression levels of SK-OV-3 and OVCAR-3 cells pretreated with IFN-γ 6 hours were detected by flow cytometry with anti-PD-L1 conjugated with APC fluorochromes. (F) SK-OV-3 GL, SK-OV-3 GL with IFN-γ pretreated (IFN-γ-SK-OV-3 GL), OVCAR-3 GL, OVCAR-3 GL with IFN-γ pretreated (IFN-γ-OVCAR-3 GL) target cells were incubated with CLDN6-CAR_1_ NK effector cells 8 hours at varying effector to target (E:T) ratios. Cell lysis was tested using a standard nonradioactive cytotoxicity assay. Error bars denote SD. ** p < 0.01, two-tailed Student's t test adjusted p value. (G) The PD-1 expression level of CLDN6-CAR1 NK cells stimulated by SK-OV-3, IFN-γ-SK-OV-3, OVCAR-3, and IFN-γ-OVCAR-3 was detected by anti-PD-1 conjugated with APC fluorochromes. (H and I) SK-OV-3 GL, IFN-γ-SK-OV-3 GL, OVCAR-3 GL, and IFN-γ-OVCAR-3 GL target cells were incubated with CLDN6-CAR_1_ NK effector cells at the indicated E:T ratios of 1:1 with or without 10 μg/mL anti-PD-L1 (H) and anti-PD-1 (I) in triplicate wells of white 96-well plates for 8 hours. Error bars denote SD. ns, not significant, * p < 0.05, ** p < 0.01, two-tailed Student's t test adjusted p value. (J) Hey GL (PD-L1 positive, Figure S7) cells were incubated with CLDN6-CAR_1_ NK cells at the indicated E:T ratios of 1:1 with or without 10 μg/mL anti-PD-L1 in triplicate wells of white 96-well plates. Target cell viability was monitored every 2 hours, 6 times in total. Error bars denote the SD. *** p < 0.001, two-way ANOVA adjusted p value. See also Figure S7-S9.

Considering the increased cytokine production and activation capacity of CLDN6-CAR_1_ NK cells and the better antitumor activities, CLDN6-CAR_1_ NK cells were applied in the following assay. Upon binding to target cells, CAR-NK cells can secrete IFN-γ and upregulate PD-L1 on target cells through the JAK/STAT pathway^34^, ^47^, ^48^. Meanwhile, target antigen stimulation induces the expression of PD-1 on the surface of CAR-NK cells^49^, ^50^. The interaction of PD-L1 to PD-1 directly inhibits the function of CAR-NK cells or indirectly enhances tumor resistance to NK cells through Treg cells promoting tumor immune escape (Fig. 6A). Western blotting and flow cytometry were used to detect the expression of PD-L1 in ovarian cancer cell lines and NK cell lines. The results showed that PD-L1 had low or no expression in SK-OV-3 and OVCAR-3 cells, moderate expression in A2780 cells, and highest expression in Hey cells. In contrast, PD-L1 was not expressed in NK and CAR-NK cells (Fig. S7A-C). We selected SK-OV-3/OVCAR-3/Hey as target cells and CLDN6-CAR_1_ NK as effector cells, and further verified that upregulation of PD-L1 expression could inhibit the killing sensitivity of CAR-NK cells to tumor cells.

Previous studies have shown that interferon-γ upregulates the expression of PD-L1 on tumor cells and promotes tumor immune escape^47^, ^48^. Since CAR-NK cells targeting CLDN6 produce interferon-γ in the process of killing OC cells, CAR-NK cells should have a similar effect on tumors and upregulate the expression of PD-L1. To test the hypothesis that CAR-NK cells can induce upregulation of PD-L1 expression on tumor cells, NK and CAR-NK cells were cocultured with SK-OV-3, OVCAR-3, A2780 and PC3 cell lines at short time and compared with non-cocultured tumor cells. The expression of PD-L1 protein was analyzed by flow cytometry and WB. The results showed that compared with parental NK cells, CAR_1_-NK cells targeting CLDN6 significantly upregulated the expression of PD-L1 on the surface of SK-OV-3 and OVCAR-3 cells and were also upregulated to some extent in the A2780 cell line. For the CLDN6-negative PC-3 cell line, PD-L1 was not significantly upregulated (Fig. 6B-6D). Flow cytometry showed that recombinant human IFN-γ induced the expression of PD-L1 in SK-OV-3 and OVCAR-3 cells, which was basically consistent with the data shown in the CAR-NK cell coculture experiment (Fig. 6E). In addition, IFN-γ did not induce the expression of PD-L1 on CAR-NK cells (Fig. S8C). Therefore, our study revealed that CAR-NK cells targeting CLDN6 can induce the expression of PD-L1 on the surface of tumor cells by secreting IFN-γ in the process of killing CLDN6-positive ovarian cancer cells.

SK-OV-3 and OVCAR-3 cells were treated with different concentrations of IFN-γ, and the results showed that the expression of PD-L1 on SK-OV-3 and OVCAR 3 cells was upregulated with increasing IFN-γ concentrations, with the highest expression at 50 ng/ml and 100 ng/ml, respectively (Fig. S8A-B). The target SK-OV-3 GL and OVCAR-3 GL cells were treated with 50 ng/ml IFN-γ and the cytotoxicity of CLDN6-CAR_1_ NK cells was tested. The results showed that the cytotoxicity of CLDN6-CAR_1_ NK cells to target cells decreased after IFN-γ stimulation (Fig. 6F). These results indicated that IFN-γ triggers the resistance of tumor cells to CAR-NK cells, which is mediated by the increased expression of PD-L1 in tumor cells. PD-L1-positive target cells were resistant to CAR-NK cells, and we speculated that one of the reasons might be the interaction between PD-1 and PD-L1. Flow cytometry analysis of NK and CAR-NK cells in the resting state showed no obvious PD-1 positive staining (Fig. S8D). However, compared with CAR-NK cells in the resting state, the positive rate of PD-1 was increased after coculture with SK-OV-3 and OVCAR-3 target cells, while the positive rate of PD-1 was increased more significantly after coculture with IFN-γ-treated target cells (Fig. 6G). After activation of CAR-NK cells by Hey with self-high expression of PD-L1, positive staining of PD-1 on CAR-NK cells was also detected, but no significant changes were observed after the addition of anti-PD-L1 monoclonal antibody (Fig. S8E).

IFN-γ-treated or untreated SK-OV-3 and OVCAR-3 cells were cocultured with CAR-NK cells by adding anti-PD-L1 or anti-PD-1 antibodies (or IgG as a control) to block PD-1/PD-L1 to enhance the cytotoxicity of CLDN6-CAR_1_ NK cells. In particular, the addition of anti-PD-L1 antibody significantly increased the sensitivity of IFN-γ-treated SK-OV-3 and OVCAR-3 cells and parental untreated SK-OV-3 cells to CAR-NK cytotoxicity (Fig.6H and 6I). To assess whether adding anti-PD-L1 can prolong the persistence of CAR-NK cells, we added the same amount of effector CAR-NK cells into PD-L1-positive Hey cells to observe the change in CAR-NK cell cytotoxicity over time. We found that the cytotoxicity of CAR-NK cells with anti-PD-L1 was more persistent than that of CAR-NK cells without anti-PD-L1 (Fig. 6J). In addition, the effect of anti-PD-L1 on tumor cells was extremely weak and mainly depended on CAR-NK cells (Fig. 6J). Flow cytometry showed that CAR-NK cells incubated with anti-PD-L1 expressed more CD107a molecules (Fig. S9A and S9B), indicating that they had a stronger degranulation ability and enhanced perforin and granzyme releasing (Fig. S9C). There was no significant difference in the detection of the cytokine IFN-γ (Fig. S9C). These results suggested that inhibition of the PD-1/PD-L1 immune checkpoint can relieve the immunosuppressive function of CAR-NK cells, prolong the persistence of cytotoxicity, and enhance the toxicity of CAR-NK cell to tumor cell.

### PD-L1 inhibitor improves the antitumor efficacy of CLDN6-CAR NK92MI cells for PD-L1^+^ subcutaneous and celiac cancers in vivo

**Figure 7:**
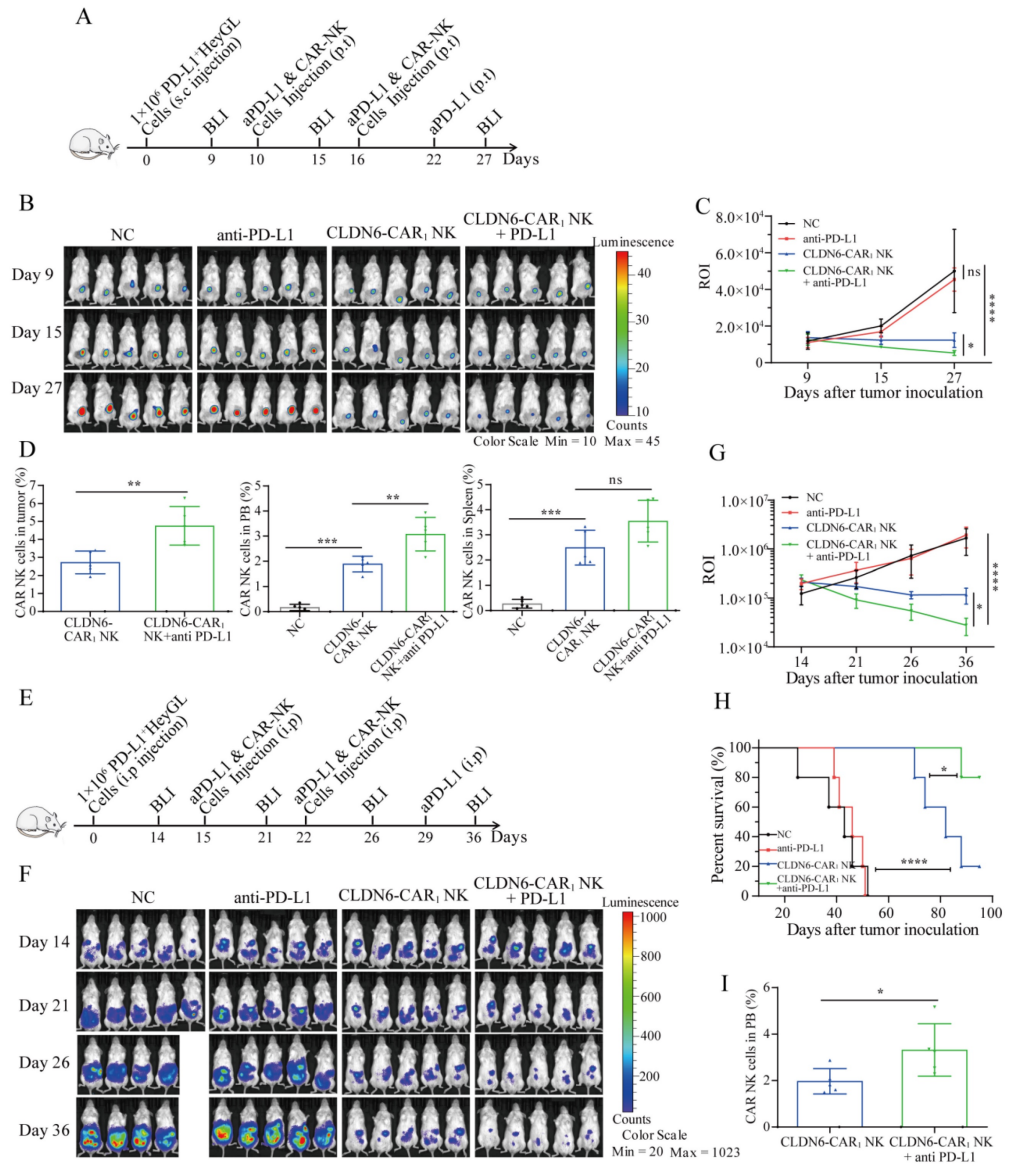
** Anti-PD-L1 enhanced the antitumor efficacy of CLDN6-CAR_1_ NK92MI cells in NSG mice.** (A) Schematic representation of the treatment scheme. NSG mice received 1 × 10^6^ Hey GL cells with subcutaneous (s.c.) injection; 5 × 10^6^ CAR-NK cells and 10mg/kg anti-PD-L1 were administered peritumorally (p.t.) at the indicated time, and BLI was performed regularly. (B and C) Representative bioluminescence images (B) and bioluminescence ROI (C) of Hey GL tumor growth in the model shown in (A) (5 mice/group). Error bars denote SD. ns, not significant, * p < 0.05, **** p < 0.001, one-way ANOVA adjusted p value. (D) Mice were euthanized 12-14 days post CAR-NK cell infusion to analyze the infiltration percentages of CAR-NK cells in tumors, PB and spleen by flow cytometry. Error bars denote SD. ns, not significant, ** p < 0.01, *** p <0.001, one-way ANOVA test adjusted p value. (E) Schematic representation of the treatment scheme. NSG mice received 1 × 10^6^ Hey GL cells with intraperitoneal (i.p.) injection; 5 × 10^6^ CAR-NK cells and anti-PD-L1 were administered intraperitoneally on the indicated time, and BLI was performed regularly. (F and G) Representative bioluminescence images (G) and bioluminescence ROI (H) of Hey GL tumor growth in the model shown in (F) (5 mice/group). Error bars denote SD. * p < 0.05, **** p < 0.0001, one-way ANOVA adjusted p value. (H) Kaplan-Meier survival curve of Hey GL cells intraperitoneally injected into mice (5 mice/group). **** p < 0.0001 (CLDN6-CAR_1_ NK group versus control group), * p < 0.05 (CLDN6-CAR_1_ NK+anti-PD-L1 group versus CLDN6-CAR_1_ NK group), Log rank (Mantel-Cox) test. (I) Infiltration percentages of CAR-NK cells in PB were analyzed by flow cytometry two weeks after CAR-NK cell infusion. Error bars denote SD. *p < 0.05, two-tailed Student's t test adjusted p value.

To further verify the efficacy of PD-L1 inhibitor in combination with CLDN6-CAR_1_ NK cells against OC in vivo, we established two different human OC xenograft mouse models. First, a subcutaneous OC model was established by injecting PD-L1^+^ Hey GL cells on day 0 (Fig. 7A). On day 9, the mice were subjected to BLI and a subcutaneous nodule signal was observed (Fig.7A and 7B). These mice were then divided into four groups: the blank group, anti-PD-L1 group, CLDN6-CAR_1_ NK group and combined therapy group (Fig. 7B). Following the flow chart in Figure 7, the results showed that PD-L1 inhibitor in combination with CLDN6-CAR_1_ NK cells induced more significant regression of Hey GL OC cells than CLDN6-CAR_1_ NK cells alone; while tumors in the blank and anti-PD-L1 groups continued to progress, as detected by BLI on days 15 and 27 (Fig. 7B and 7C). Interestingly, CAR-NK cell infiltration in the combination group was higher than that in the CLDN6-CAR_1_ NK cell group, as detected by flow cytometry (Fig. 7D).

Next, we constructed another mouse model in which PD-L1^+^ Hey GL cells were intraperitoneally injected into mice, which mimicked clinical tumor metastasis patterns of OC (Fig. 7E and 7F). The effect of CAR-NK cells combined with anti-PD-L1 was significantly better than that of each monotherapy and it also prolonged the survival time of mice, indicating that the combination of PD-L1 inhibitor and CAR-NK cells has a synergistic antitumor effect (Fig. 7F-7H). On day 14 after CAR-NK cells infusion, CAR-NK cells were also detected in peripheral blood and the proportion in the anti-PD-L1 combination with CAR-NK group was higher than that in the CAR-NK group, suggesting that anti-PD-L1 prolonged the survival time of CAR-NK cells in vivo (Fig. 7I). These results suggested that the addition of anti-PD-L1 treatment enhanced the in vivo antitumor activity of CLDN6-CAR_1_ NK cells.

## Discussion

Identification of a safe and effective specific tumor antigen is the key to CAR cell therapy^20^. CD19 CAR T cell therapy for B cell lymphoma or lymphocytic leukemia is the most widely studied clinical indication and is a successful FDA-approved CAR cell therapy^9^, ^51^. However, the selection of targets for CAR cell therapy in solid tumors needs to be further explored. CLDN6, one of the 27 members of the claudin family, is mainly involved in the tight connection of epithelial cell membranes and intercellular adhesion playing an important role in the occurrence and development of tumors^22^. In this study, immunohistochemistry and western blot assays demonstrated that CLDN6 was significantly upregulated in ovarian cancer tissues and a variety of ovarian cancer cell lines and localized in cell membranes. Moreover, CLDN6 antigen was not detected in human normal vital organ tissues, which is consistent with relevant reports^28^, ^52^. Therefore, CLDN6 may be an ideal target for CAR-NK cell therapy in ovarian cancer.

Compared with the second-generation CAR molecules, the third-generation CAR has two costimulatory domains, which can further improve the signal transduction function so that CAR-NK cells have better in vivo amplification ability and longer-lasting activity^53^. Therefore, in this study, we chose NKG2D as transmembrane domain and 2B4/DAP10 as costimulatory domains to construct third-generation CAR_1_-NK cells targeting CD19 (as control) or CLDN6. It is well known that each specific domain can promote the activity of CAR-NK cells, thus promoting antigen-induced NK cell-mediated antitumor cytotoxicity. Obviously, it is reasonable and feasible to construct the CAR domain by the self-activated receptor of NK cells^14^, ^54^. In addition, we also constructed CD28 as transmembrane domain and CD28/4-1BB as the costimulatory domain, that is, the classical T cell CAR molecular domain, to construct CAR_2_-NK cells targeting CLDN6. The results of functional activation and antitumor activity of CAR-NK cells constructed with different domains showed that CLDN6-CAR_1_ NK cells constructed with self-activated receptors as domains had stronger activation and specific killing ability, which was similar to that reported by Kaufman's team^14^. In our experiment, cytokine secretion and CD107a expression were basically consistent with the killing effect of CAR-NK cells constructed by different domains, which fully proved that the new CAR_1_-NK cells targeting CLDN6 have accurate and efficient targeting specificity and that the self-activating elements NKG2D, 2B4 and DAP10 play an important role in activating the function of CAR-NK cells^18^, ^55^.

Although the molecular interaction between NK cells and target cells has received close attention, few studies have focused on analyzing the heterogeneity of the behavior of individual NK cells^41^. Standard cytotoxicity analysis usually provides information on population average, while cytotoxicity analysis based on living cell imaging can evaluate the characteristics of individual cells, and different NK cell-target cell interaction patterns can be thoroughly assessed^40^. Cell migration is the result of physical constraints (such as hardness, porosity and ordering), substrate interactions (such as integrin-mediated adhesion) and chemical signals from the local microenvironment (such as chemokines). These are mechanically integrated into membrane- and cytoskeleton-mediated cell movements, such as adhesion, traction, protuberance, deformation and polarization^56^, ^57^. Migration behavior can reflect the level of NK cell activation^41^.

In the in vitro experimental system of CLDN6-targeted CAR-NK cells cocultured with CLDN6-positive SK-OV-3 cells, membrane extension during the migration of CAR-NK cells was observed by live cell imaging. A more accurate analysis of membrane extension shows that the lamellar projection at the leading edge of the cell is lamellipodium, and the filamentous structure is adhesive filamentous pseudopodia, which is similar to the results reported by Marta Mastrogiovanni et al^58^, indicating that the introduction of CAR molecules into NK cells changes cellular behaviors such as polarization, migration and extension of NK cells. In particular, CAR-NK cells targeting CLDN6 with self-activated receptors showed more obvious behavioral changes, which proved that NKG2D, 2B4 and DAP10 play an important role in activating CAR-NK cells^18^. However, live cell imaging showed that most of the parental NK cells and CAR-NK cells targeting CD19 were round in shape, the short membrane extension was short pseudopodia and round protuberance, or valvular extension or vesicular process, and lamellipodium with filamentous pseudopodia were rarely seen. With the passage of time, patchy and filamentous pseudopodia could also be seen in some cells, which may be formed due to continuous stimulation of NK cell activation by antigen, and could eventually kill a small number of target cells, which is consistent with the relevant research reports^41^. Co-culture of CAR-NK cells with CLDN6-negative PC-3 cells observed similar phenomena to parental NK and CD19-targeted CAR-NK cells, which fully demonstrated the precise targeting ability of CAR-NK cells targeting CLDN6 constructed by us.

Interestingly, we accidentally found that CDLN6-targeted CAR-NK cells constructed by self-activating elements have the ability to kill continuously, which was similar to the results reported by Bruno Vanherberghen et al^41^ that a small number of activated NK cells have the ability to continuously kill multiple target cells and are called "serial killers", indicating that our CAR-NK cells have a faster activation ability, thus inducing the death of target cells more quickly. Similarly, K. Reinhard et al^28^ also mentioned the continuous killing ability of CAR-T cells. At the same time, the CAR-NK cells targeting CDLN6 constructed by self-activating elements had the characteristics of shorter killing time and fewer CAR-NK cells needed to kill more cancer cells. These results suggest that CAR-NK cells have precise targeting and high killing efficiency and may also be related to the amount of perforin rapidly released by CAR-NK cells^41^. We observed the morphological changes and killing time of NK cells through real-time dynamic imaging of living cells. The results showed that CLDN6-CAR_1_ NK cells constructed by self-activation elements had stronger cell activation and killing ability, and these modifications to our CAR-NK cells will be tested in future works to further improve the efficacy against clinical ovarian cancer.

In this study, a human ovarian cancer cell line was used to construct subcutaneous tumors to verify the efficacy of CLDN6-targeted CAR NK cells. The results showed that CLDN6-targeted CAR NK cells had good antitumor effects in mice and were well tolerated in vivo, thus demonstrating the efficacy and safety of CLDN6 as a target antigen for CAR NK cell therapy. Systemic delivery of NK cells via vein may sometimes lead to severe autoimmune toxicity, while regional delivery methods such as intra-artery or intra-tumor can prevent severe systemic exposure and nontarget toxicity^59^. A clinical trial (NCT01837602) evaluated the safety and feasibility of intratumoral CAR-T cells for the treatment of metastatic breast cancer, where intratumoral CAR-T cells were well tolerated and induced inflammatory responses within the tumor^60^. Multiple preclinical studies in NSG mice demonstrated that peritumoral injection of CAR-T/NK cells successfully inhibited tumor progression in vivo, superior to intravenous injection, and that peritumoral administration strategies improved effector cell infiltration in tumor tissues^43^, ^61^. Our in vivo data also confirmed that peritumoral administration of CAR-NK cells directly increased the number of CAR-NK cells infiltrating the tumor, showing a good antitumor effect. Studies have shown that intraperitoneal injection of CAR-T cells in the treatment of abdominal tumors is superior to systemic infusion^62^, and thoracic infusion of CAR-T cells in the treatment of malignant pleural diseases also achieves good therapeutic effects, due to conducive to earlier contact and activation of CAR-T cells with target antigens, thus enabling timely acquisition of proliferation and differentiation^17^, ^63^. Therefore, to improve the activity of CAR-NK cells, we used the intraperitoneal administration method to treat the ovarian cancer model with peritoneal metastasis. The results showed that intraperitoneal injection of CAR-NK cells targeting CLDN6 achieved good antitumor effects and prolonged the survival of mice. This may be related to the earlier and more effective accumulation of CAR-NK cells in tumors by intraperitoneal injection^63^. In the model of hematogenous metastasis, bioluminescence could only be detected in the liver, which may be related to the biological characteristics of ovarian cancer and the host, such as solid nodules in the liver, strong fluorescence signals, cystic metastases in the lungs, few solid components and weak signals. Therefore, we speculated that the weak signal in the lung may be masked by the strong fluorescence signal in the liver, which leads to no fluorescent signal in the chest or other metastatic lesions during imaging.

According to the latest research, genetic susceptibility genes cause cancer not only by increasing the probability of gene mutation but also by shaping immune function; impaired migration and adhesion of immune cells in tumor patients may lead to inefficient antitumor immunity^58^. Studies have shown that the number of infiltrating immune cells in ovarian cancer tissues of patients with high expression of CLDN6 is significantly reduced^64^. These factors may explain why immune checkpoint inhibitors are not as effective in the treatment of ovarian cancer. Since infusion of CAR-T or NK cells alone may be immunosuppressed by the PD-1/PD-L1 axis in the tumor microenvironment, thus affecting the therapeutic effect, in this study, we tested a new combination immunotherapy therapy, namely, CAR-NK cells combined with immune checkpoint inhibitors. The results showed that the combination of the two has a synergistic effect, hoping to provide a new treatment strategy for comprehensive anticancer therapy in the future.

Ovarian cancer is a typical immunosuppressive tumor. PD-L1 is highly expressed in most ovarian cancers and their microenvironment. Although our understanding of PD-L1 expression regulation is not complete, it can be determined that PD-L1 regulation mainly works through the classical type II interferon pathway JAK1/2-STAT1/3-IRF1 axis and the PD-L1 axis, and NK cells are an important source of IFN-γ in the tumor microenvironment^47^, ^48^. Based on numerous reports and our results, it can be speculated that CAR-NK cells secrete a large amount of IFN-γ after binding to target cells, and tumor cells react quickly to IFN-γ secreted by activated NK cells, which binds to interferon receptors and then activates JAK1, JAK2 and STAT1 in tumor cells, in turn upregulating the expression of PD-L1 on tumor cells (Figure 6A). In this study, we investigated immune escape from CAR-NK cytotoxicity after upregulation of PD-L1 in ovarian cancer. We observed that immune escape from CAR-NK cytotoxicity was associated with high levels of PD-L1 expression in tumor cells. This is similar to the conclusion that PD-L1 expression is upregulated on tumor cells following T cell-mediated immune escape^65^, ^66^.A large number of studies have shown that NK cells can also express the inhibitory receptor PD-1, so immune checkpoint inhibitors may increase the killing effect of NK cells against PD-L1+ tumors^67^-^69^. The importance of immune checkpoints beyond T cells in a review of NK cells possibly play an important role in the antitumor response of immune checkpoint blocking, suggesting that the PD-1 axis is important for NK cells in the tumor microenvironment^49^. It is worth noting that PD-1-negative NK cells may not be able to kill tumor cells because they fail to activate or have become impotent, and NK cells are both phenotypically and functionally heterogeneous^70^. The expression of PD-1 may not be detected in quiescent NK cells, and it can be induced and upregulated in the activated state, but this does not represent cell failure. Only the interaction with PD-L1 in the tumor microenvironment can provide inhibitory signals and reduce the function of NK cells, which can be reversed by anti-PD-L1 antibodies. In fact, functional impairment of NK cells appears to be mainly due to the serious imbalance between inhibitory and triggering signals, reflecting upregulation or downregulation of different receptors.

In a study of continuous stimulation of CAR-NK-92 cells by target cells, Oelsner et al.^50^ reported that the expression of PD-1 was significantly increased after continuous activation of NK-92/63.z and NK-92/63.28.z cells but not NK-92/63.137.z cells. It is worth noting that the killing activity of NK-92/63.z and NK-92/63.28.z CAR cells is significantly better than that of NK92/63.137.z. Our results were similar to those above reported by Oelsner et al.^50^, suggesting that PD-1 upregulation in CAR-NK cells may be related to NK cell killing activity. CAR-NK cells targeting CLDN6 have specific targeting and are highly activated, and once stimulated by antigen, the inhibitory receptor, mainly PD-1, may be more easily upregulated than the parental NK cells^71^. After coculture of CAR-NK cells with target cells, it seems that the induction of PD-1 in cells appears to be clinically significant because it confirms that there may be a closer PD-1/PD-L1 interaction between PD-L1^+^ ovarian cancer and CAR-NK cells (Figure 6A). This may explain why blocking the PD-1/PD-L1 checkpoint could increase the cytotoxicity of CAR-NK cells to IFN-γ-stimulated ovarian cancer cells (PD-L1^+^ cells) in our research. Our results are consistent with reports that NK cells combined with PD-L1 inhibitors have good antitumor effects against the background of PD-L1^+^ tumor cells^48^, ^49^, ^72^. In addition, the number of infiltrating CAR-NK cells increased after the addition of anti-PD-L1 in our study, and anti-PD-L1 prolonged the persistent activity of CAR-NK cells in tumors, which was consistent with related reports^73^. It was also confirmed that anti-PD-L1 relies on CAR-NK cells to function^31^, ^49^. PD-L1 on tumor cells can also interact with PD-1 on T cells, thus promoting the expansion of Treg cells. Treg cells have been known to inhibit the function and persistence of NK cells^34^, ^74^ and blocking PD-1/PD-L1 may prevent the induction of Treg cells.

In view of the translational value of CLDN6 CAR-NK cells, we preregistered it on the clinical trial website (NCT05410717) last year, hoping to recruit patients for clinical trials to evaluate the safety and preliminary efficacy of claudin-6 targeting CAR-NK cells in patients with claudin-6 positive advanced solid tumors (ovarian cancer and others).

However, our research is not without limitations. We did not use in situ ovarian cancer or a patient-derived xenograft tumor-bearing mouse model to study the antitumor activity of CAR-NK cells in vivo. This study lacks the specific molecular mechanism of CAR-NK cell activation, so it is suggested to further explore the molecular mechanism of CAR-NK cell action to provide a theoretical basis for the optimization of CAR-NK cell structure and function in the future. In this study, severely immunodeficient mice lacking a complete immune system were used, making it difficult to evaluate the effect of the autoimmune system on CAR-NK cell activity in vivo. We did not use knock-out CLDN6 cell lines to verify its targeting in vivo and in vitro. If there are conditions in the future, we will try to verify it in this way, which will be more convincing to the targeting of CAR-NK cells in this study.

## Conclusion

We have successfully designed CLDN6-CAR NK92MI cells and demonstrated its feasibility and efficacy in ovarian cancer in vitro and in vivo. CAR-NK cells combined with immune checkpoint inhibitors, anti-PD-L1, could synergistically enhance the antitumor efficacy of CLDN6-targeted CAR-NK cells. Our findings may provide a promising therapeutic approach in the clinical application of immunotherapy against ovarian cancer.

## Supplementary Material

Supplementary figures and table.

Supplementary videos.

## Abbreviations

NK: natural killer
CAR: chimeric antigen receptor
CLDN6: claudin-6
OC: ovarian cancer
GVHD: graft-versus-host disease
CRS: cytokine release syndrome
ITSM: immune receptor tyrosine-based switching motif
scFv: single chain variable fragment
FACS: fluorescence-activating cell sorting
WB: western blot
PVDF: polyvinylidene fluoride
IHC: immunohistochemistry
qPCR: quantitative real-time polymerase chain reaction
AGE: agarosegel electrophoresis
GAPDH: glyceraldehyde-3-phosphate dehydrogenase
ELISA: enzyme-linked immunosorbent assay
IFN-γ: interferon-γ
TNF-α: tumor necrosis factor-α
GM-CSF: granulocyte-macrophage colony stimulating factor
BLI: bioluminescence imaging
TAAs: tumor-associated antigens
DAP10: DNAX-activating protein 10
GFP: green fluorescent protein
TM: Transmembrane
