# Supplementary material for: NK-92MI Cells Engineered with Anti-claudin-6 Chimeric Antigen Receptors in Immunotherapy for Ovarian Cancer
Source: Int J Biol Sci. 2024 Feb 11;20(5):1578–601. doi: 10.7150/ijbs.88539 (PMC10929190; doi:10.7150/ijbs.88539)
Supplement: Supplementary file 1 — Supplementary figures and table. [file ijbsv20p1578s1.pdf]

# Supplementary file for
# NK-92MI cells engineered with anti-claudin-6 chimeric antigen receptors in immunotherapy for ovarian cancer

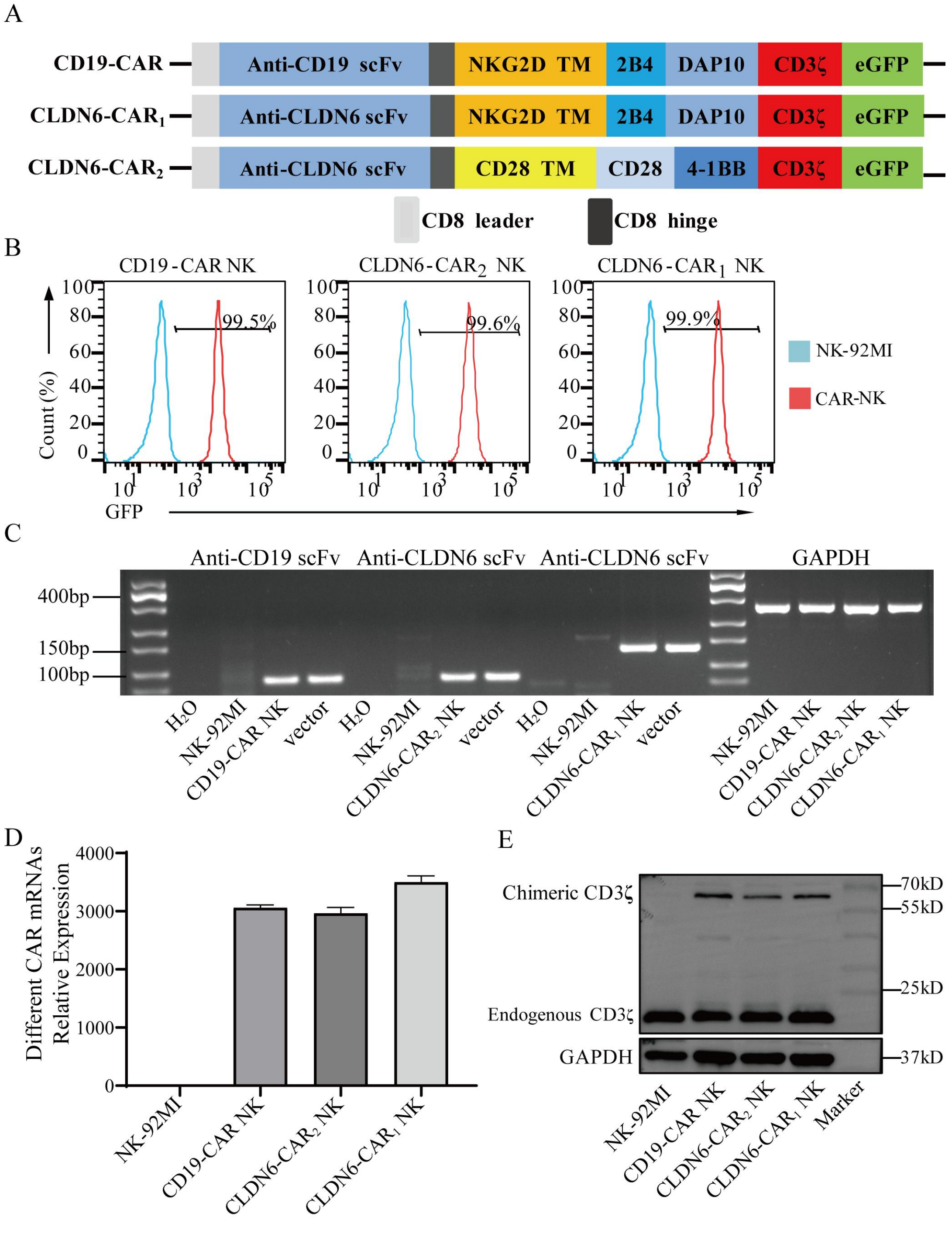


**Figure S1. Construction and characterization of CLDN6-specific CARs.**

(A) Schematic representation of CD19- and CLDN6-targeted CARs. CD19-CAR cells were used as a control.

(B) The percentages of CD19- and CLDN6-CAR-transduced CAR-NK92MI cells were detected by flow cytometry. eGFP served as a marker of CAR molecule expression.

(C) The expression levels of different CAR mRNAs were detected by agarose gel electrophoresis. $H_2O$ was used as a negative control, and lentiviral vectors of different CAR molecules were used as a positive control. GAPDH was used as an internal reference gene.

(D) Relative expression of different CAR molecule mRNAs normalized to the GAPDH gene in CAR-NK92MI cells was assessed by qPCR.

(E) Different CAR expression in CAR-NK92MI cells was detected by western blot analysis. A CD3ζ-specific antibody was used to detect endogenous and chimeric CD3ζ. See also Figure S2 and S3.

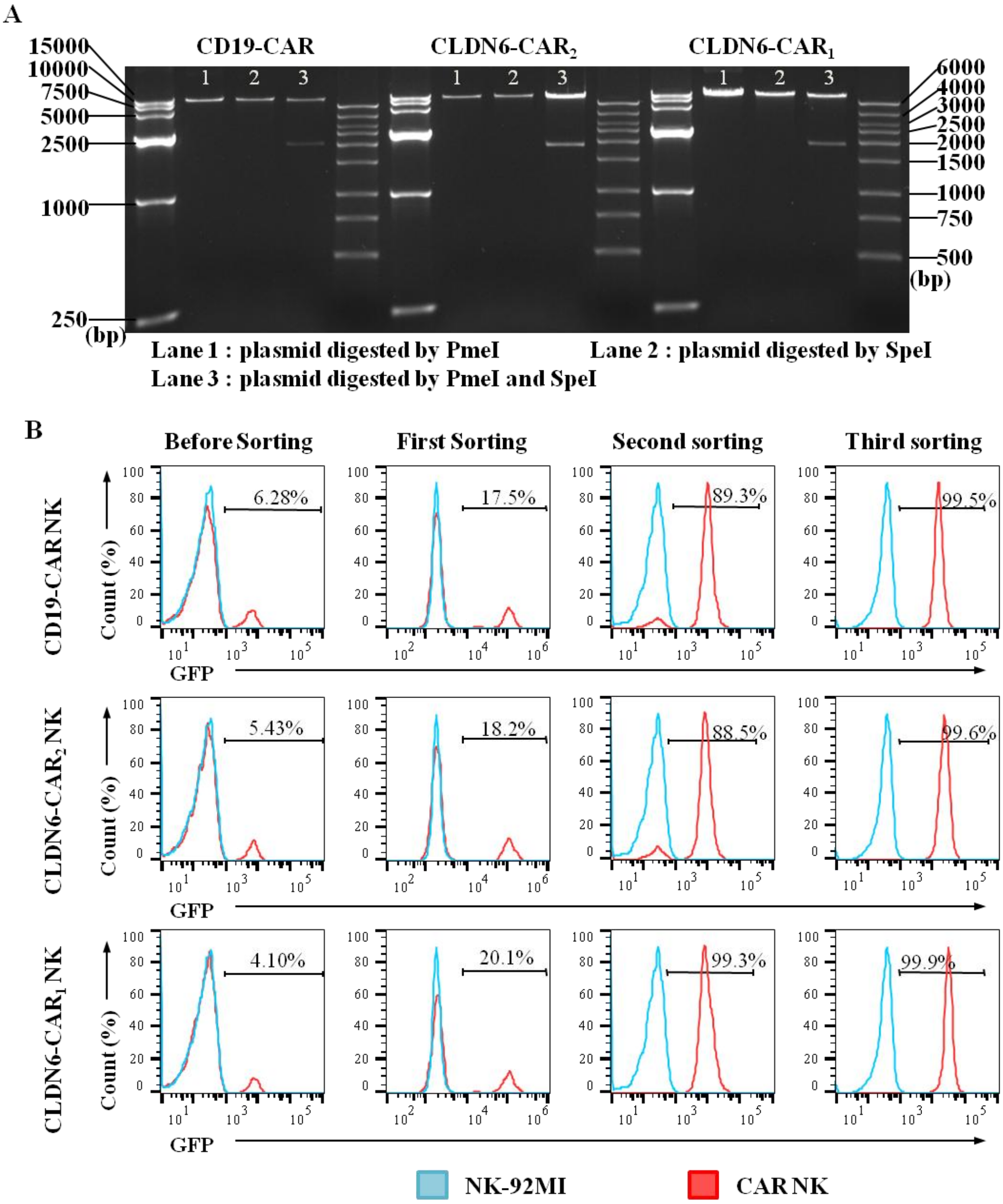


**Figure S2. Structural verification of lentiviral vectors and flow cytometry sorting (FCMS).**

(A) After digestion of the CAR lentiviral vector plasmid by restriction endonucleases PmeI and SpeI, the CAR gene was detected by agarose gel electrophoresis (AGE).

(B) Flow cytometry sorting (FCMS). eGFP served as a marker.

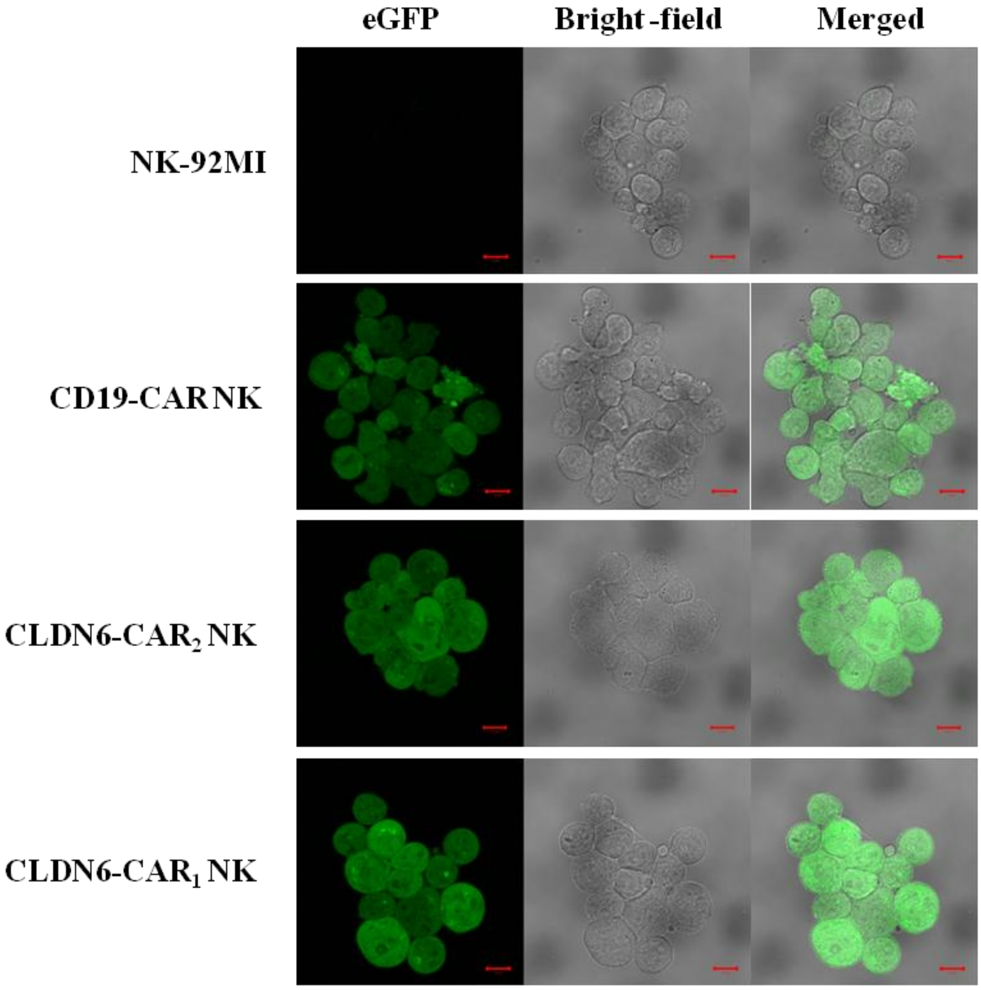


**Figure S3. The morphology and GFP expression of CAR-NK92MI cells were observed by confocal microscopy.** Scale bars, 10 μm.

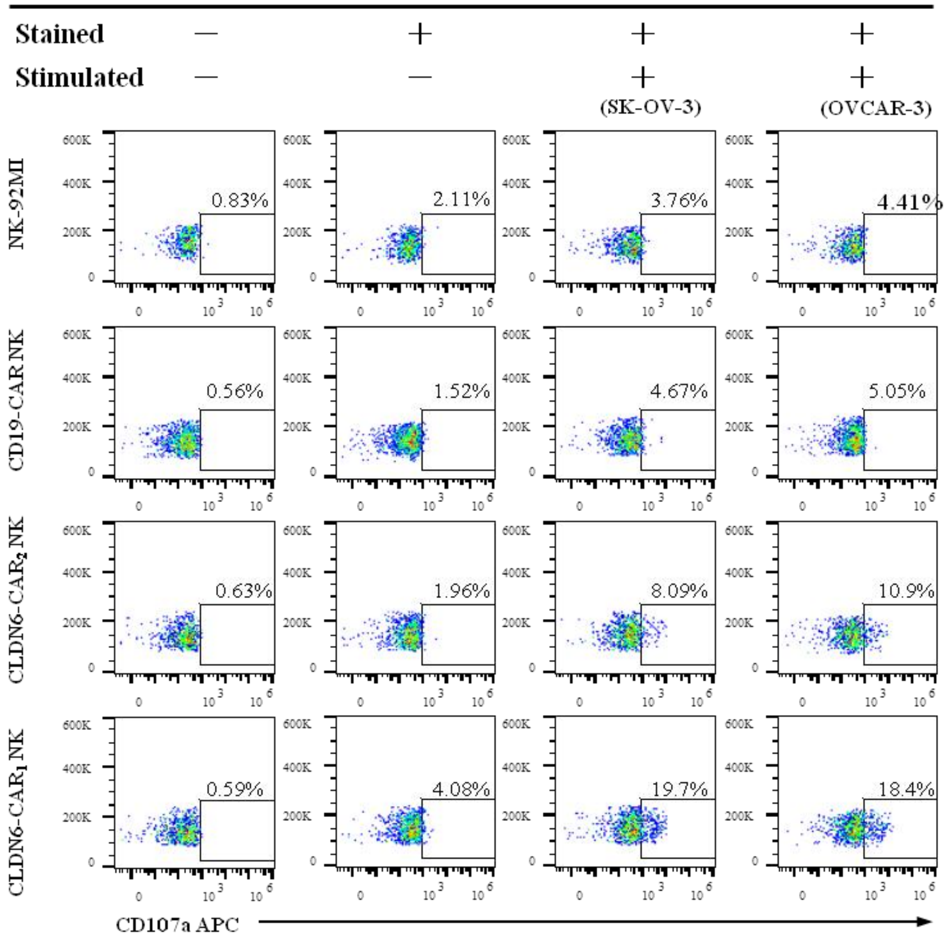


**Figure S4. CD107a detection on the CAR-NK92MI cell surface.** Detection of the cell surface activation marker CD107a on CAR-NK cells after stimulation with SK-OV-3 and OVCAR-3 cells at an E:T ratio of 1:1 or no stimulation. Flow cytometry using Abs specific to human CD107a conjugated with APC fluorochromeswas performed.

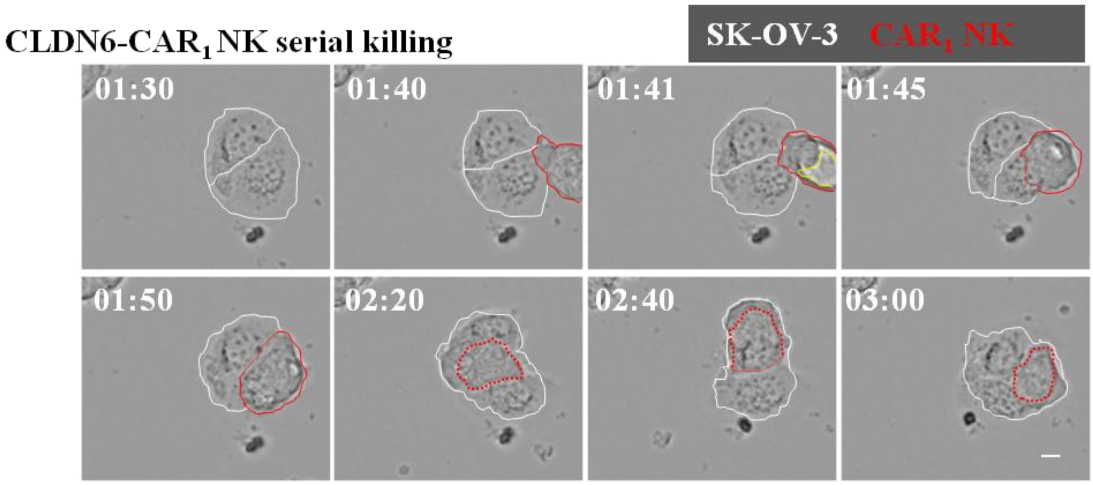


**Figure S5. Representative time-lapse images of CLDN6-CAR$_1$ NK92MI serial killing (yellow lines) and SK-OV-3 (red lines)**. In the same field of view indicated in Figure 3, the CAR-NK cells killed one target cell and then moved on to kill another. See also Figure 3.

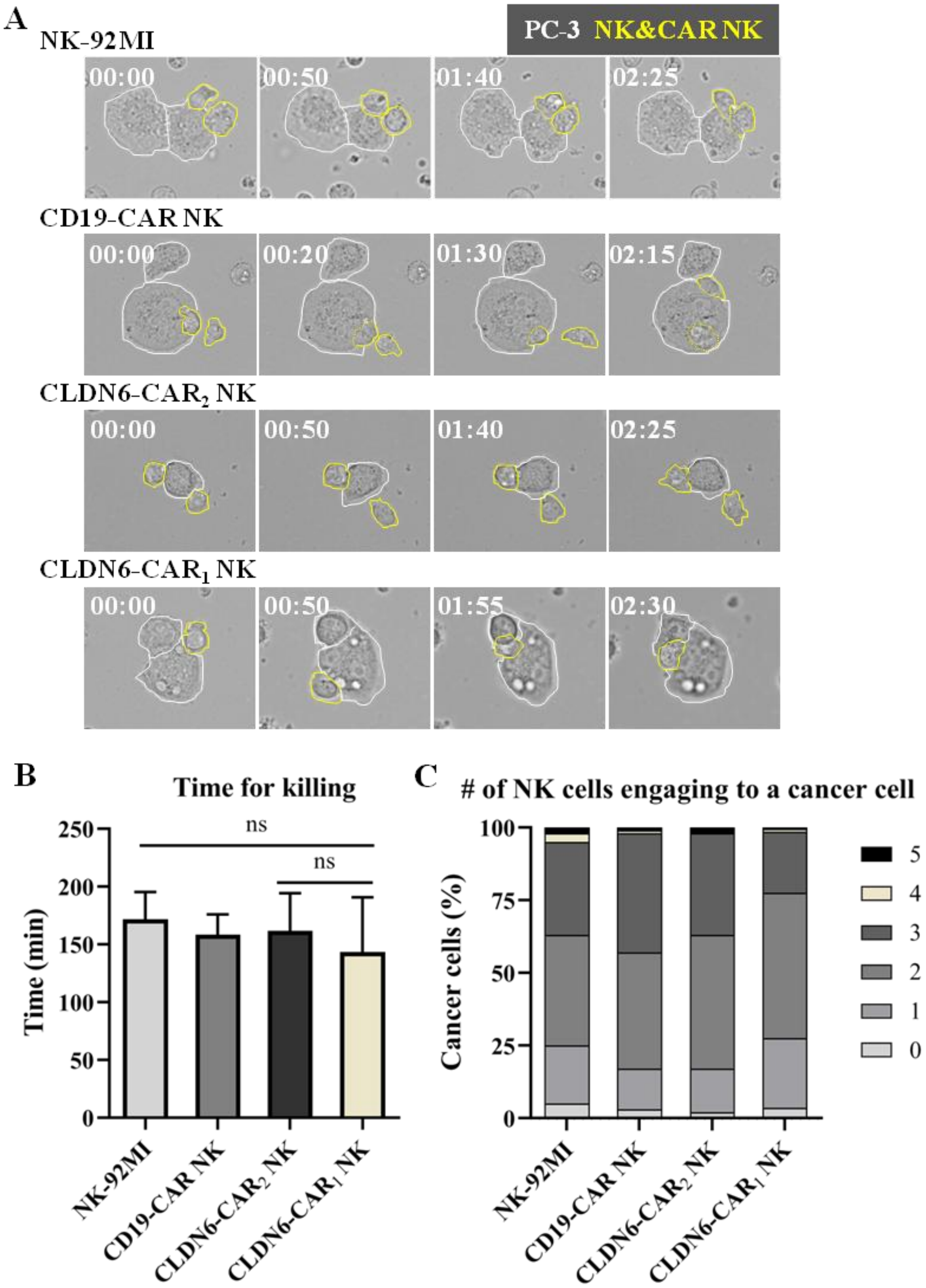


**Figure S6. Analysis of the interaction between CAR-NK92MI cells and PC-3 (CLDN6-negative) cytotoxicity based on live-cell imaging**

(A) Representative time-lapse images of the interaction between different CAR-NK cells (yellow lines) and PC-3 cells (white lines).

(B–C) Time for killing (B) and the number of NK cells engaging with a cancer cell (C). Error bars denote SD. ns, not significant, Mann-Whitney test adjusted p value.

A time display on the left upper corner indicated in (A) represents the changed time post CAR-NK cell coculture with target cells.

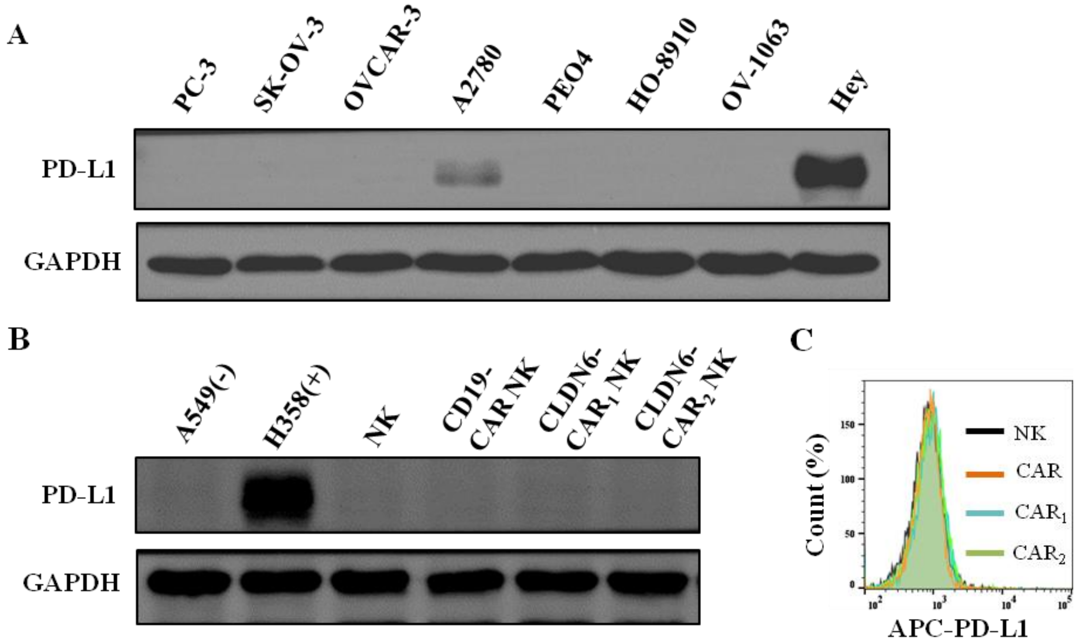


**Figure S7. PD-L1 expression in all ovarian cancer cell lines and NK-92MI cells was detected by western blot (A and B) or flow cytometry (C).** A549 cells were used as a negative control, and H358 cells were used as a positive control.

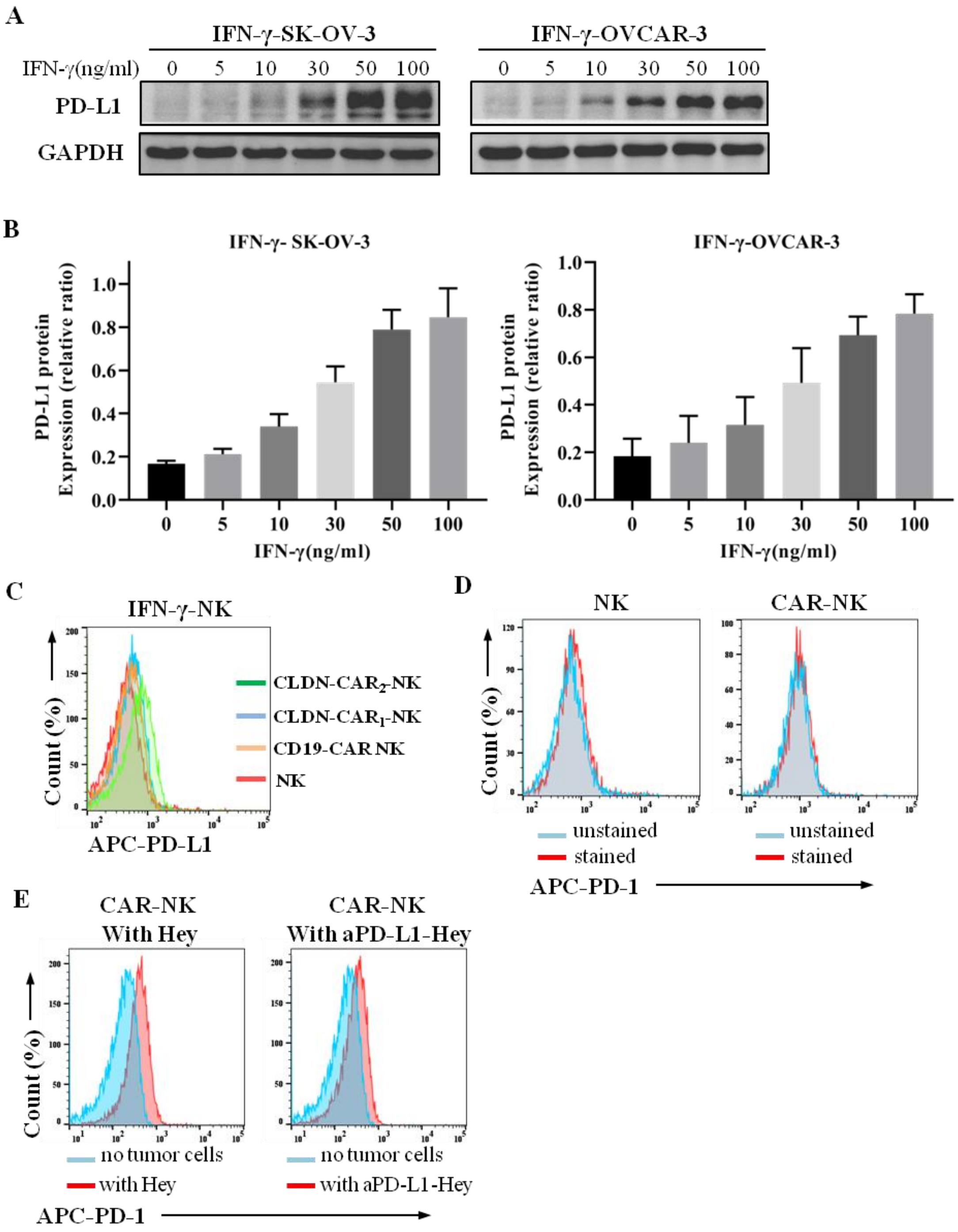


**Figure S8. PD-1/PD-L1 was detected by western blotting and flow cytometry.**

(A and B) SK-OV-3 and OVCAR-3 cells were treated with different concentrations of IFN-γ (0, 5, 10, 30, 50, and 100 ng/ml), and then the expression of PD-L1 was detected by western blot (A) and analyzed by ImageJ software (B). Error bars denote SD.

(C) CD19-CAR, CLDN6-$CAR_1$, and CLDN6-$CAR_2$ NK cells treated with IFN-γ were examined by flow cytometry with Abs specific to human PD-L1 conjugated to APC fluorochromes.

(D) PD-1 detection in the resting state of NK and CLDN6-$CAR_1$ NK cells with flow cytometry.

(E) The PD-1 expression level of CLDN6-$CAR_1$ NK cells stimulated by Hey was detected by anti-PD-1 conjugated with APC fluorochromes.

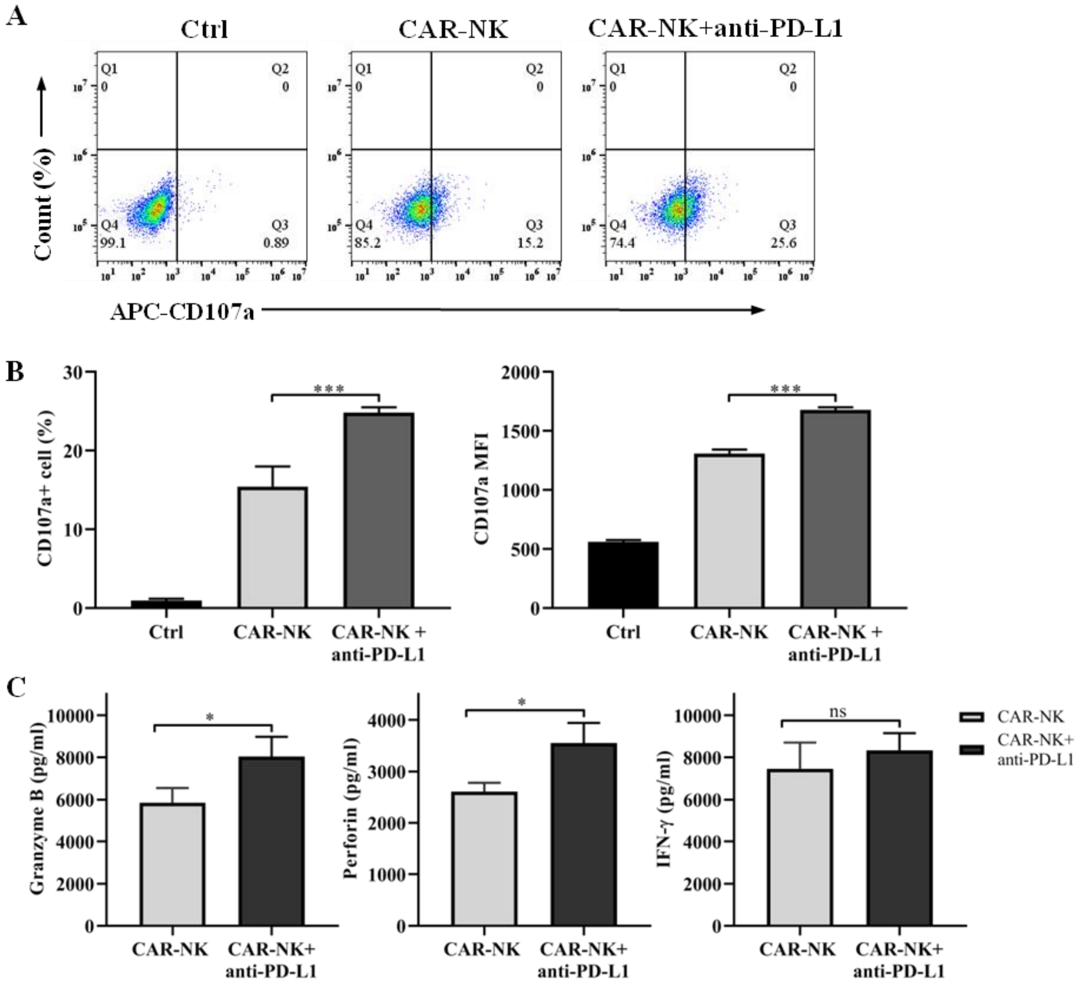


**Figure S9. Activation and cytokine secretion of CLDN6-CAR$_1$ NK92MI cells combined with anti-PD-L1.**

(A) Detection of the cell surface activation marker CD107a on CLDN6-CAR$_1$ NK cells combined with anti-PD-L1 via FACS.

(B) The CD107a+ percentage of NK and CAR-NK cells and fluorescence intensity were analyzed. Error bars denote SD. *** $p < 0.001$, one-way ANOVA with Holm-Sidak test adjusted p value.

(C) The secretion of IFN-γ, granzyme B, and perforin by CAR-NK cells combined with anti-PD-L1 therapy was determined by enzyme-linked immunosorbent assay (ELISA). Error bars denote SD. ns, not significant, * $p < 0.05$, two-tailed Student's t test adjusted p value.

**Table 1. Determination of lentivirus titers with different CAR molecules.** Unit (TU/ml).

| | CD19-CAR | CLDN6-CAR1 | CLDN6-CAR2 |
|---|---|---|---|
| **Titer (TU/ml)** | **$2.40\times10^8$** | **$2.31\times10^8$** | **$1.81\times10^8$** |
